# Bacterial Biofilm Eradication Agents: A Current Review

**DOI:** 10.3389/fchem.2019.00824

**Published:** 2019-11-28

**Authors:** Anthony D. Verderosa, Makrina Totsika, Kathryn E. Fairfull-Smith

**Affiliations:** ^1^Institute of Health and Biomedical Innovation, Queensland University of Technology, Brisbane, QLD, Australia; ^2^School of Biomedical Sciences, Queensland University of Technology, Brisbane, QLD, Australia; ^3^School of Chemistry, Physics, and Mechanical Engineering, Queensland University of Technology, Brisbane, QLD, Australia

**Keywords:** antibiotics, bacteria, biofilm, biofilm antibiotic tolerance, biofilm eradication agent, infection, resistance

## Abstract

Most free-living bacteria can attach to surfaces and aggregate to grow into multicellular communities encased in extracellular polymeric substances called biofilms. Biofilms are recalcitrant to antibiotic therapy and a major cause of persistent and recurrent infections by clinically important pathogens worldwide (e.g., *Pseudomonas aeruginosa, Escherichia coli*, and *Staphylococcus aureus*). Currently, most biofilm remediation strategies involve the development of biofilm-inhibition agents, aimed at preventing the early stages of biofilm formation, or biofilm-dispersal agents, aimed at disrupting the biofilm cell community. While both strategies offer some clinical promise, neither represents a direct treatment and eradication strategy for established biofilms. Consequently, the discovery and development of biofilm eradication agents as comprehensive, stand-alone biofilm treatment options has become a fundamental area of research. Here we review our current understanding of biofilm antibiotic tolerance mechanisms and provide an overview of biofilm remediation strategies, focusing primarily on the most promising biofilm eradication agents and approaches. Many of these offer exciting prospects for the future of biofilm therapeutics for a large number of infections that are currently refractory to conventional antibiotics.

## Introduction

Biofilm formation is a significant virulence mechanism in the pathogenesis of many medically important bacterial pathogens, such as *Pseudomonas aeruginosa* (Gellatly and Hancock, 2013), *Staphylococcus aureus* (Gordon and Lowy, 2008), and *Escherichia coli* (Beloin et al., 2008). The number of diseases being attributed or associated with biofilm infections is large, with some common examples including vaginitis (Machado et al., 2016), colitis (von Rosenvinge et al., 2013), conjunctivitis (Behlau and Gilmore, 2008), gingivitis (Vieira Colombo et al., 2016), urethritis (Delcaru et al., 2016), and otitis (Post, 2001). In fact, it is estimated that ~80% of all microbial infections in humans are a direct result of biofilms (Davies, 2003). One biofilm-related infection of particular medical concern is *P. aeruginosa* biofilms in the lungs of cystic fibrosis patients. This opportunistic pathogen has been known to cause acute and chronic lung infections that can result in significant morbidity and mortality (Wagner and Iglewski, 2008). A second area of considerable concern is that of chronic wound infections. Highly persistent biofilm-related wound infections, which commonly involve the pathogens *P. aeruginosa* and *S. aureus* (Omar et al., 2017), are suggested to be responsible for over 80% of the 100,000 limb amputations carried out on diabetic patients in each year (James et al., 2008). An additional area of importance when considering biofilm-related infection is implanted medical devices. Microbial adhesion resulting in biofilm formation on implanted medical devices is a common occurrence and can lead to serious illness and death (Habash and Reid, 1999). These implanted medical devices, which can include intravascular catheters, urinary catheters, pacemakers, heart valves, stents, and orthopedic implants, are commonly used to saves lives but can present a significant health risk when colonized by bacterial biofilms (Francolini and Donelli, 2010).

Most antimicrobial treatments available are generally developed and evaluated against microorganisms in the planktonic (free-living) mode of life. Consequently, these treatments are often ineffective against pathogenic biofilms (Costerton et al., 1987; Lebeaux et al., 2014), which can be up to one thousand times more tolerant to antimicrobial treatments (Stewart and William Costerton, 2001; Luppens et al., 2002; Davies, 2003). The phenomenon of biofilm recalcitrance makes them extremely difficult to treat and eradicate effectively. Thus, new strategies for the prevention, dispersal and treatment of bacterial biofilms are urgently required. This review presents an overview of bacterial biofilm development and the current methods used to prevent, disperse, and treat bacterial biofilms, with a particular focus on the development of novel biofilm eradication strategies.

## Biofilm Formation

**Figure 1:**
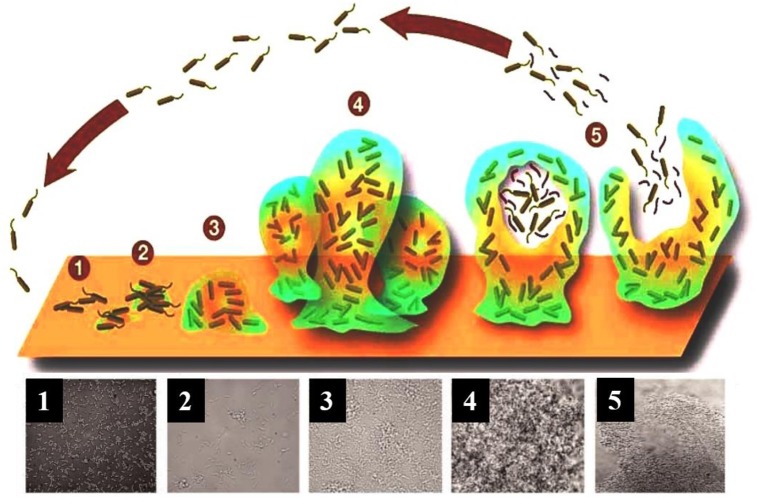
A model showing the typical stage-wise development of a bacterial biofilm accompanied by transmitted light microscopy images showing these different stages for a *P. aeruginosa* biofilm. Republished with permission of Annual Reviews, Inc. (Stoodley et al., 2002); permission conveyed through Copyright Clearance Center, Inc.

Biofilms are complex three-dimensional communities of microorganisms adhering to a surface and encased in a protective exopolymeric substance. Biofilm formation progresses over five main stages (Figure 1). In stage one, individual planktonic cells migrate and adhere to a surface. Providing the correct conditions are present, these adherent cells then initiate biofilm production on the surface and become encased in small quantities of exopolymeric material. In stage two, adherent cells exude an extracellular polymeric substance (EPS) and become irreversibly attached to the surface, which results in cell aggregation and matrix formation. In stage three, the biofilm begins to mature by developing microcolonies and water channel architecture, while also becoming significantly more layered. In stage four, the fully mature biofilm reaches its maximum cell density and is now considered a three-dimensional community. In stage five, the mature biofilm releases microcolonies of cells from the main community, which are free to migrate to new surfaces spreading the infection to other locations (Stoodley et al., 2002; Schachter, 2003).

## The Extracellular Polymeric Substance (EPS)

The extracellular matrix encasing the cells in a biofilm, also referred to as the EPS, is composed of a complex mixture of proteins, lipids, nucleic acids (extracellular-DNA), and polysaccharides (Annous et al., 2009). These constituents not only assist in securing the biofilm to the surface, but also trap nutrients, provide structural support, and shield against host immune responses and antimicrobial treatments (Flemming et al., 2007). In addition to the above functions, the EPS is also responsible for holding the community of biofilm cells in close proximity, thereby enabling cell-to-cell communication (quorum sensing), and facilitating the exchange of genetic material through horizontal gene transfer (Hausner and Wuertz, 1999).

## Cell-to-cell Communication (Quorum Sensing)

**Figure 2:**
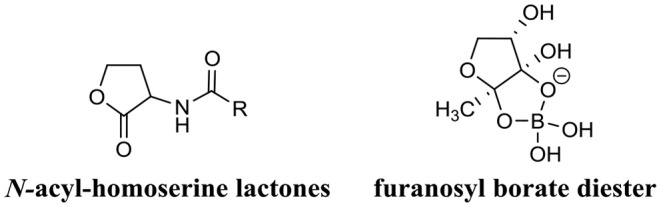
Chemical structure of two predominant types of small molecule autoinducers involved in quorum sensing.

**Figure 3:**
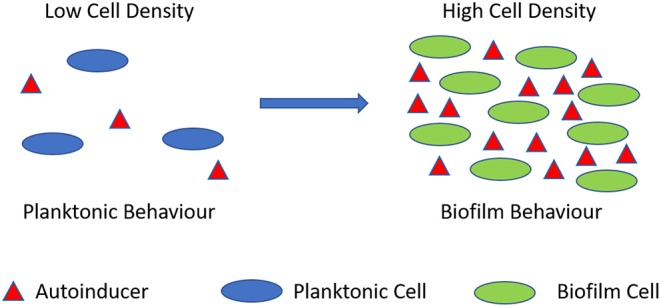
Quorum sensing illustration. During planktonic cell growth (blue ovals), the relative amount of autoinducers (red triangles) is proportionally low. As cells enter a densely populated mode of growth (green ovals) the relative proportion of autoinducers increases.

Biofilms are known to control their population density through a cell-to-cell signaling mechanism known as quorum sensing (Schachter, 2003). Cell-to-cell communication is a complex regulatory process which prevents biofilm cell density from reaching an unsustainable level (Nadell et al., 2008). Quorum sensing is reliant on signaling molecules known as autoinducers (Figure 2). These autoinducers are constantly being produced by the bacterial cells, and thus, as cell density increases, so does the level of autoinducers (Figure 3). At a specific cell density, a critical threshold concentration of autoinducers is reached, which is known as the quorum level (Annous et al., 2009). During this time, autoinducer receptor binding leads to the repression or activation of several target genes. This modulation of the quorum sensing process allows bacteria to display a unified response that benefits the entire bacterial community by maintaining the optimal biofilm size and co-ordinating virulence phenotypes (Nadell et al., 2008; Annous et al., 2009; Dickschat, 2010). This unified response allows the biofilm to behave more like a multicellular organism, which enables the bacterial community to adapt to changing environmental conditions. The benefit of quorum sensing is not limited to controlling population density. In fact, quorum sensing has also been shown to aid the spread of beneficial mutations throughout the biofilm colony, enhance access to nutrients, and contribute to antibiotic tolerance (Hannan et al., 2010).

## Biofilm Antibiotic Tolerance (BAT)

Bacteria in biofilms are inherently more tolerant to antimicrobial treatment when compared directly to planktonic cells of the same strain. In fact, studies have shown that bacteria growing in biofilms are often thousands of times more tolerant to antimicrobial treatment than their planktonic counterparts (Stewart and William Costerton, 2001; Luppens et al., 2002; Davies, 2003). While, the mechanisms of antibiotic resistance in planktonic bacteria are generally well-understood (Munita and Arias, 2016), those same mechanisms (mutations, efflux pumps, and antibiotic modifying enzymes) do not appear to be the main cause of biofilm-mediated antibiotic tolerance. For example, inherently drug-susceptible bacterial strains often exhibit significant antibiotic tolerance when in the biofilm mode of life, however, when biofilm-residing cells are dispersed (released) from the main community, antimicrobial susceptibility is quickly restored for these cells (Anderl et al., 2000). Thus, biofilm antibiotic tolerance (BAT) is thought to involve alternative mechanisms to bacterial antimicrobial resistance.

**Figure 4:**
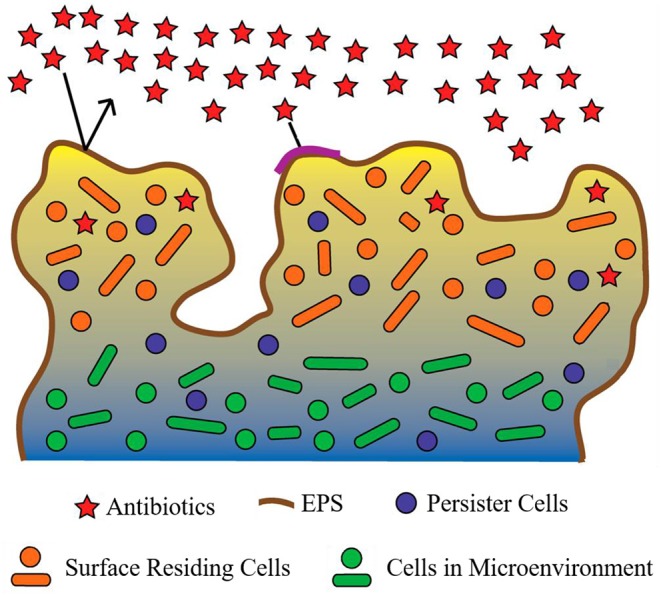
Proposed mechanisms contributing to biofilm antimicrobial tolerance (BAT). The biofilm shown is comprised of bacteria (circles and ovals), which are encapsulated by the extracellular polymeric substance (EPS) (dark-brown line surrounding biofilm and multi-colored background within biofilm). Red stars represent antibiotics which are in contact with the biofilm. Restricted penetration of antibiotics through the biofilm EPS is depicted by the black arrows (indicating antibiotics failing to penetrate the EPS of the biofilm) and the red stars at the surface of the biofilm (indicating antibiotics that have failed to diffuse past surface-residing cells). Orange circles and ovals surrounded with yellow/tan background represent surface-residing cells which are in contact with the antibiotics (red stars). Green circles and ovals surrounded by a blue/gray background are indicative of microenvironments within the biofilm (areas of reduced oxygen concentration and reduced cell replication). Purple circles indicate persister cells present within the biofilm (small subpopulation of cells within the biofilm that enter a protected metabolically quiescent state recalcitrant to the action of antimicrobials). Image adapted from Penesyan et al. (2015).

BAT has been defined as the ability of biofilm-residing bacteria to survive antimicrobial treatment by utilizing their existing complement of genes (Anderson and O'Toole, 2008). BAT can be grouped into two categories: innate (resulting from growth in a biofilm) and induced (resulting as a response to antimicrobial treatment). Several major innate factors have been identified which directly influence BAT (Costerton et al., 1999; Lewis, 2001; Donlan and Costerton, 2002; Dunne, 2002; Stewart, 2002; Hoiby et al., 2010) and are briefly discussed below (Figure 4).

## Innate Factors Mediating BAT

### Restricted Penetration

The EPS of a biofilm has long been considered the major contributor to BAT (Donlan and Costerton, 2002). However, the supporting evidence for this is conflicting. The EPS of several biofilm-forming species have demonstrated an innate ability to prevent antibiotic penetration (Campanac et al., 2002; Davenport et al., 2014). However, this phenomenon is not conserved between the EPS of all biofilm-forming species and also appears to be antibiotic specific. For example, ciprofloxacin and ampicillin were found to effectively penetrate and diffuse through *Klebsiella pneumoniae* biofilms, ultimately reaching distal cells (Anderl et al., 2000). Furthermore, ciprofloxacin also exhibited similar penetration and diffusion activity in *P. aeruginosa* biofilms (Walters et al., 2003). Likewise, tetracycline was able to effectively reach all cells within *E. coli* biofilms (Stone et al., 2002). Interestingly, many of these antibiotics are still ineffective at eradicating the biofilm. While restricted penetration may be a major contributing factor of BAT for some antibiotics in some biofilms, its effects are certainly not universal. Thus, additional or complementary mechanisms that facilitate BAT also exist.

### Reduced Growth Rate

While restricted pentation does not always explain BAT, reduced growth rate appears to play a far more evident role in BAT. The complex internal structure of a biofilm is known to produce microenvironments, which are deprived of oxygen and nutrients (Brown et al., 1988). Deprivation of oxygen and nutrients are well-established cues for slowed bacterial growth and antimicrobial resistance in many species (Brown et al., 1988; Field et al., 2005). Considering most antibiotics target rapidly replicating bacterial cells, it is of little surprise that areas of slow-growing or dormant cells within a biofilm would be unaffected by antibiotics and thus exhibit high levels of antibiotic tolerance. Several studies have demonstrated a direct link between biofilm microenvironments, which produce slow-growing cells and BAT (Anderl et al., 2003; Walters et al., 2003; Borriello et al., 2004). However, not all antimicrobial agents require rapidly replicating cells to facilitate their mode of action, and many of these agents are still highly tolerated by biofilms, for example, chlorine in the treatment of multi-species biofilms (Barraud et al., 2009). Thus, slow bacterial growth alone is not sufficient to confer BAT either.

### Persister Cells

Persister cells represent a minute subpopulation of bacterial cells, which exist in a dormant state and exhibit extreme antimicrobial tolerance (Wood et al., 2013). The presence of persister cells within a bacterial population is not a recent discovery; in fact, their existence was first described as early as 1942 (Hobby et al., 1942). Early studies discovered that when a planktonic population of *S. aureus* cells was treated with penicillin, ~1% of the cells were not killed. Two years later, Bigger supported this finding and documented that one out of a million *S. aureus* cells was not killed by treatment with penicillin (Bigger, 1944). Furthermore, Bigger also determined that these surviving cells, which he termed persisters to differentiate them from resistant mutants, had not undergone a genetic alteration, but instead were simply a phenotypic variant that was tolerant to antibiotics. Despite their early discovery, the role of persister cells in bacterial pathogenesis remained largely unexplored until the study of bacterial biofilms uncovered the significant role of persister cells in BAT (Lewis, 2010).

Unlike planktonic bacterial populations the presence of persister cells within a biofilm community affords them protection from elimination by the immune system, and despite their small numbers, their contribution to pathogenesis becomes more significant in biofilm infections (Lewis, 2005, 2010). Several studies have now demonstrated that after treating a biofilm with antibiotics, a small population of persister cells will remain regardless of the concentration of antibiotic utilized (Spoering and Lewis, 2001; Harrison et al., 2005a,b). Once the treatment ceases and the antibiotic concentration decreases, these remaining persister cells can act as a nucleation point to repopulate the biofilm, ultimately producing a relapsing biofilm infection (Lewis, 2001). Interestingly, the majority of these repopulated biofilm residing cells exhibit no additional antimicrobial tolerance or resistance compared to the original cells that were eradicated, strongly supporting that the persister state is a phenotypic variant rather than a mutation.

The universal presence of persister cells within biofilms is perhaps the most plausible innate mechanism of BAT described so far, and while persisters do not harbor antimicrobial resistance genes directly, they certainly provide a perfect platform for the development of resistant mutants. Consequently, many research groups have focused their efforts on investigating the mechanisms of persister cell formation (Keren et al., 2004a,b; Spoering et al., 2006), with the hope that their findings will enable the development of antibacterial agents which can target and eradicate these fascinating cells.

## Induced BAT Mechanisms

The mechanisms of induced BAT appear to be more complicated than the innate factors contributing to this phenomenon and are less well-understood with only a few studies on the topic (Bagge et al., 2004a,b; Szomolay et al., 2005; Redelman et al., 2014; Zhao et al., 2015). Antimicrobial treatment represents a significant stress signal for biofilm-residing cells, and consequently, it is reasonable to assume that antimicrobial treatment could select biofilm-specific antimicrobial resistance genes, and these genes would contribute to BAT. An interesting example of how antibiotics can induce such a response in biofilms is the effect that some antibiotics have on EPS production. Ziebuhr et al. documented how administering sub-inhibitory concentrations of several common antibiotics to *Staphylococcus epidermidis* biofilms activated the expression of the *ica* gene cluster, which mediates the production of polysaccharide intercellular adhesin (PIA), a vital factor for *S. epidermidis* biofilm formation (Rachid et al., 2000). Young et al. and Hoiby et al. found similar effects, albeit with different genes in *E. coli* and *P. aeruginosa*, respectively (Sailer et al., 2003; Bagge et al., 2004b). While these examples have not specifically been linked to BAT the idea that biofilm residing bacteria may regulate the expression of specific genes in response to antimicrobials to facilitate BAT certainly appears plausible.

## Biofilm Inhibition Strategies

The material matrix of implanted medical devices and biomaterials provide an ideal site for bacterial adhesion promoting mature biofilm formation (Arciola et al., 2012). Thus, methods which prevent bacterial attachment to these materials represent an obvious preventative strategy. The most common method for preventing bacterial adhesion is surface modification. Here, the exterior surface of the implanted medical device or biomaterial is altered, either directly or with the aid of a coating, to produce a barrier which is inhospitable to bacteria (Bazaka et al., 2012). This strategy has shown significant promise for preventing biofilm-related infections resulting from orthopedic implants (Arciola et al., 2012). Thus, the area of surface modification to prevent biofilm formation is a large field, and many comprehensive reviews on this topic already exist (Katsikogianni and Missirlis, 2004; Arciola et al., 2012; Bazaka et al., 2012; Campoccia et al., 2013).

The use of small molecule biofilm inhibitors is another approach used to prevent biofilm formation. In fact, the anti-biofilm properties of a biofilm inhibitor are often employed to passivate the surface of an implanted medical device or biomaterial (Nablo et al., 2005; Boase et al., 2018). The use of biofilm inhibitors is one of the largest areas in biofilm remediation research with a plethora of unique biofilm inhibitors currently described (e.g., phenols, imidazoles, furanone, indole, bromopyrrole, etc.) (Rabin et al., 2015). As such, there are many comprehensive reviews on the topic of biofilm inhibition agents (Simões et al., 2010; Worthington et al., 2012; Rabin et al., 2015).

## Biofilm Dispersal as a Treatment Strategy

Biofilm dispersal agents generally interfere with chemical pathways or processes, such as quorum sensing, which are required for bacteria to maintain the biofilm mode of existence (McDougald et al., 2012). As disperser cells are generally more susceptible to antimicrobial treatment than biofilm-residing cells, this strategy has recently become an intense area of study. Consequently, a variety of new and promising biofilm dispersal agents have been discovered and reviewed by others (Fleming and Rumbaugh, 2017; Guilhen et al., 2017; Roy et al., 2018). While promising, the use of biofilm-dispersal agents as a treatment strategy can be problematic, as disperser cells, if left untreated, are likely to translocate and seed infection in new areas, ultimately spreading the initial infection. Hence, most dispersal agents are utilized as a combined treatment where the dispersal agent is co-administered with an antimicrobial agent (Marvasi et al., 2014; Reffuveille et al., 2015). Co-treatment generally involves administering a combination of drugs concurrently, in this case, a biofilm dispersal agent and an antibiotic, to exert a synergistic effect. While co-administering a dispersal agent with an antibiotic has yielded some promising results *in vitro* (Barraud et al., 2006; Reffuveille et al., 2015; Roizman et al., 2017), this treatment strategy can be challenging to translate in the clinic, as ensuring that both agents are present at the target site in the correct concentration is often difficult (Fleming and Rumbaugh, 2018). Furthermore, drug co-administration treatments are often associated with several challenges, including complex treatment schedules, increased risk of adverse effects, increased treatment costs, and antagonism (Rybak and McGrath, 1996; Tamma et al., 2012). Consequently, standalone treatments, such as the development of biofilm eradication agents (BEAs) have become an attractive option.

## Biofilm Eradication Agents

BEAs are antibiotics which can target and eradicate biofilm-residing cells as a standalone treatment. The design and discovery of BEAs constitute an emerging area in biofilm remediation research. A variety of promising BEAs have already been developed, and their activity, design, and potential uses are reviewed below.

## Antimicrobial Peptides

Antimicrobial peptides (AMPs) are one of the most well-studied classes of BEAs and are often considered an attractive alternative to antibiotics (Baltzer and Brown, 2011). They are ubiquitous compounds, produced in a variety of plant, invertebrate, and animal species. AMPs can vary greatly in size (between five to over ninety amino acids) and molecular mass (between 1 and 5 kDa). They are most commonly cationic in nature (overall positive charge), which has led to them being referred to as cationic antimicrobial peptides (Brown and Hancock, 2006); however, anionic forms have also been reported (Harris et al., 2009). Their antimicrobial mechanism of action is still not fully understood, but their activity is often linked to cytoplasmic membrane disruption and inhibition of protein folding or enzyme activity (Shai, 1999; Bechinger and Gorr, 2017). While the potential use of AMPs as an alternative to antibiotics has received a great deal of attention over the past several decades, their use against microbial biofilms is far most recent.

**Figure 5:**
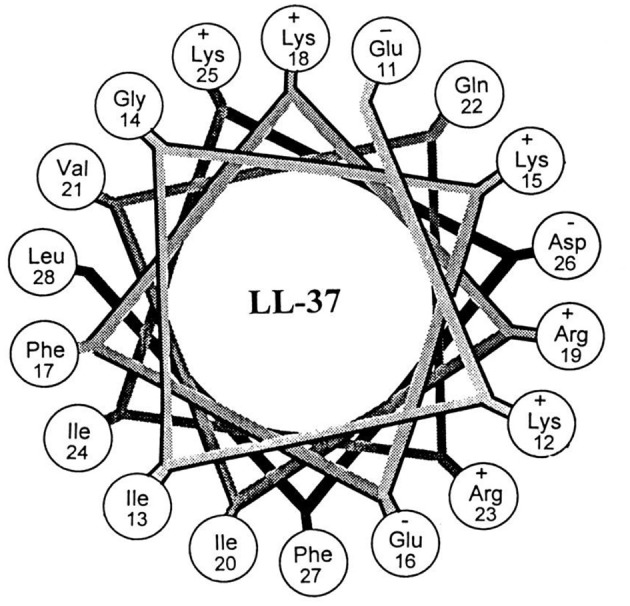
Helical wheel illustration of residues 11–28 of the mature LL-37. Republished with permission of American Society for Microbiology (Turner et al., 1998); permission conveyed through Copyright Clearance Center, Inc.

LL-37 (Figure 5) was one of the first AMPs reported as possessing the potential for biofilm eradication (Overhage et al., 2008). LL-37 is a human cathelicidin-derived broad spectrum AMP, which is amphipathic and found in most bodily fluids (Burton and Steel, 2009; Nijnik and Hancock, 2009). Hancock first reported that low concentrations (0.11 μM) of LL-37 were able to decrease *P. aeruginosa* cell attachment to plastic surfaces, while higher concentrations (0.9 μM) reduced the overall thickness of established biofilms (40% reduction in thickness) (Overhage et al., 2008). In a subsequent study by Cohen, LL-37 was found to eradicate *P. aeruginosa* biofilms in an *in vivo* animal model at a concentration of 556 μM (Chennupati et al., 2009). Interestingly, in a separate study by Marchini, LL-37 was also shown to exhibit anti-biofilm activity against the Gram-positive pathogen *Staphylococcus epidermidis* with low concentrations (0.22 μM) preventing cell attachment and higher concentrations (0.22–7.12 μM) preventing mature biofilm establishment (Hell et al., 2010). While the study did not directly examine *S. epidermidis* biofilm eradication by LL-37, similarities in its biofilm inhibition concentrations with *P. aeruginosa* would suggest that its eradication activity is likely to be broad-spectrum. In a more recent study by Li and co-works, LL-37 was found to exhibit potent *S. aureus* biofilm eradication activity (Kang et al., 2019). LL-37 was able to significantly eradicate *S. aureus* biofilm residing cells (>4-log reduction in CFU) (Kang et al., 2019). LL-37 certainly appears to exhibit many of the characteristics of a promising BEA, it has both Gram-negative and Gram-positive efficacy, and low human cell toxicity (Gordon et al., 2005), however, its use as a BEA remains limited. Instead, LL-37 seems to function better as a biofilm inhibitor (Overhage et al., 2008) rather than a true BEA. Nevertheless, the potential of this AMP has undoubtedly been demonstrated, and hopefully investigations into its use as a BEA will continue.

**Figure 6:**
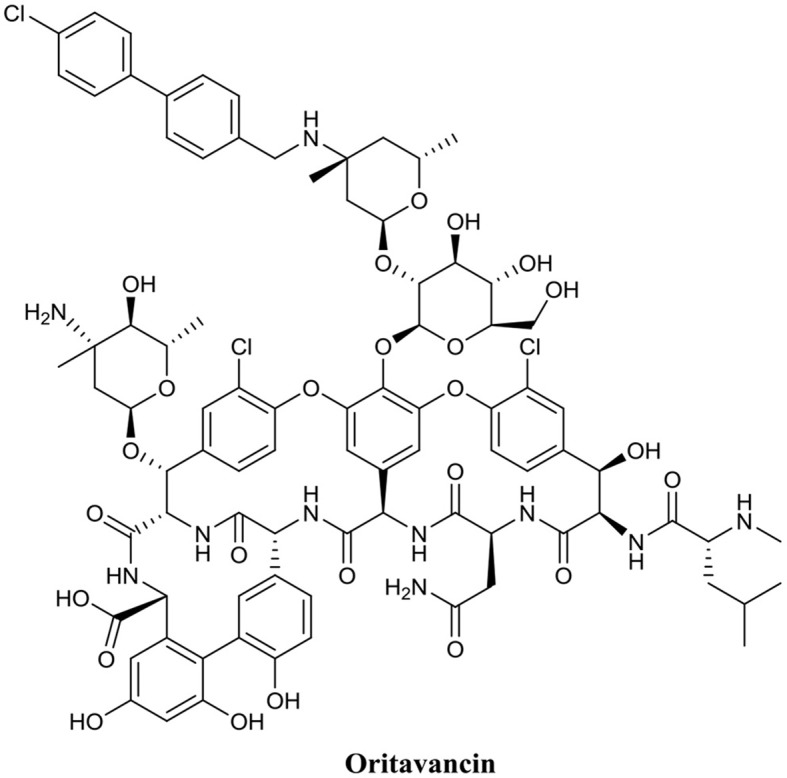
Chemical structure of Oritavancin.

Another AMP with promising biofilm eradication activity is oritavancin (Figure 6). Oritavancin is a semi-synthetic lipoglycopeptide, which has been developed for the treatment of medically problematic Gram-positive infections, such as methicillin-susceptible *S. aureus* (MSSA), methicillin-resistant *S. aureus* (MRSA), and vancomycin-resistant *S. aureus* (VRSA) (Allen, 2010). Moeck and coworkers demonstrated that oritavancin possessed both impressive planktonic and biofilm eradication activity (Belley et al., 2009). Oritavancin was able to completely eradicate (99.9%) MSSA, MRSA, and VRSA biofilms at concentrations between 0.3 and 4.5 μM. Most importantly, the concertation required to completely eradicate established biofilms were within one doubling dilution of the respective concertation required to kill planktonic cells of the same strain. Thus, it appears that the activity of oritavancin is not significantly diminished by the formation of a biofilm. Interestingly, oritavancin, which is a structurally related analog of vancomycin, is significantly less toxic to humans than other lipoglycopeptides, such as vancomycin and telavancin (Darpo et al., 2010). This property along with the high potency of this AMP certainly suggests that oritavancin is a promising BEA, at least against Gram-positive pathogens.

While several other AMPs have exhibited some level of biofilm eradication activity (Wei et al., 2006; Beckloff et al., 2007; Hou et al., 2010), most AMPs appear to exhibit more potent anti-biofilm action (inhibition or dispersal) than biofilm eradication activity (Overhage et al., 2008; Flemming et al., 2009; Hou et al., 2009). Consequently, many AMPs are often utilized in combination with antimicrobial treatment (Dashper et al., 2005; Eckert et al., 2006). For example, G10KHc, a novispirin G10 derived AMP, acted synergistically with tobramycin when administered as a co-treatment against *P. aeruginosa* biofilms (Eckert et al., 2006). Considering the promising anti-biofilm properties of these compounds and their demonstrated synergistic effect with antimicrobials, AMPs represent one promising avenue for the development of treatments for biofilm-related infections. However, as their inherent structures are often quite large and complicated, compared to other antibiotic classes, such as fluoroquinolones and beta-lactams, their modification, development and utilization as BEAs may ultimately be limited.

## Quaternary Ammonium Compounds

Quaternary ammonium compounds (QACs) are a large class of broad-spectrum bactericidal agents. Their core structure is amphiphilic comprising a hydrophobic alkyl chain and a hydrophilic quaternary ammonium group, and they are often referred to as AMP mimics, however, their structures are far simpler. Their activity is associated with disruption of the bacterial plasma membrane, which leads to metabolite leakage, and eventual cell lysis (Ioannou et al., 2007). The antibacterial activity of this class of compounds is well-documented, and consequently, many of these types of compounds are already in common commercial use as antiseptics, disinfectants, and preservatives (Russell, 2003). However, their use as BEAs has only recently been explored.

**Figure 7:**
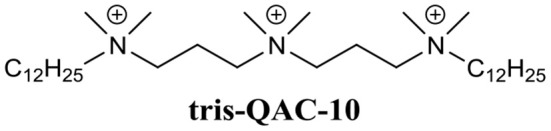
Chemical structure of tris-QAC-10.

Wuest et al. developed a variety of mono-, bis-, and tris-QACs and demonstrated biofilm eradication activity against pre-formed *S. aureus* and *E. faecalis* biofilms (Jennings et al., 2014). In particular, tris-QAC-10 (Figure 7) was able to completely eradicate established biofilms of *S. aureus* at 50 μM, and *E. faecalis* at 25 μM (Jennings et al., 2014). While tris-QAC-10 also exhibiting potent planktonic activity against *E. coli* and *P. aeruginosa* (MIC 0.5 and 1 μM, respectively), its biofilm eradication activity against these Gram-negative species was not investigated (Jennings et al., 2014). The reported QACs exhibited significant eukaryotic cell toxicity, with authors noting that the development of less toxic analogs was currently underway (Jennings et al., 2014). In a follow-up publication by the same group, a set of multiQACs was reported that not only exhibited impressive biofilm eradication activity (complete eradication of *S. aureus* biofilms at 25 μM) but were also considerably less toxic compared to earlier QACs (Forman et al., 2016). Considering the development of these compounds as BEAs is relatively recent, their potency and spectrum of activity are highly impressive. Providing that human cell toxicity can be reduced further in subsequent derivatives, these compounds are certainly one of the more promising approaches for the development of BEAs.

**Figure 8:**
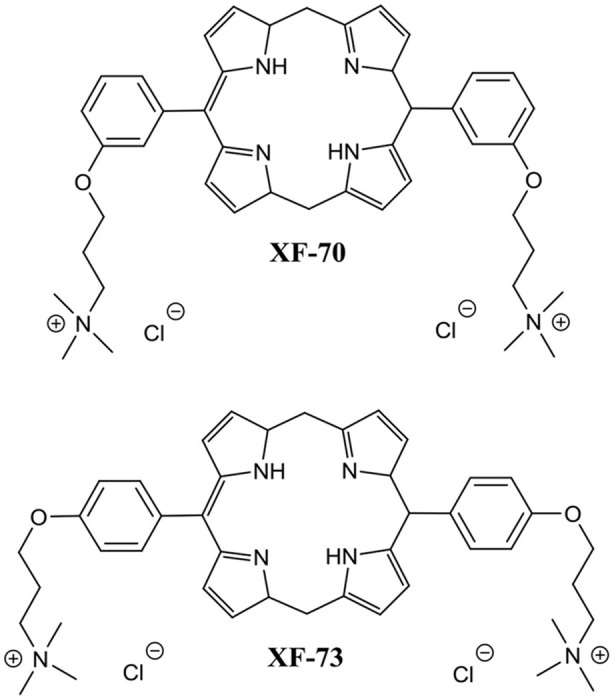
Chemical structures of XF-70 and XF-73.

Another recent study reported on two unique dicationic porphyrin QACs XF-70 and XF-73 (Figure 8) with demonstrated potent planktonic antibacterial activity (Farrell et al., 2010). Chopra et al. evaluated these two QACs for biofilm eradication activity against *S. aureus* biofilms (Ooi et al., 2010). Both XF-70 and XF-73 completely eradicated pre-formed *S. aureus* biofilms at a concentration of only 2.6 μM. In addition, XF-70 and XF-73 were also compared to a diverse panel of commonly administered antimicrobial agents and were found to be >128-fold more potent than all other tested agents under the same assay conditions against the same *S. aureus* strain. Furthermore, in a subsequent study by Love et al., XF-73 demonstrated a remarkably low propensity for inducing bacterial resistance (Farrell et al., 2011). With the impressive properties of these QACs there is little surprise that at the time of writing Destiny Pharma has already completed and passed phase 1 clinical trials with XF-73 (Yendewa et al., 2019).

The biofilm eradication properties of QACs position them as a promising strategy for the treatment of biofilm-related infections, however, their inherent toxicity is still a hurdle which will need to be overcome or may limit their clinical use to mostly topical treatments. Furthermore, much like AMPs, the BEA activity of QACs appears to be far more conducive to the treatment of Gram-positive pathogens as opposed to Gram-negative ones. However, as their structures are considerably less complicated and smaller than AMPs, the potential to synthetically modify their core structures to enhance Gram-negative activity is certainly a more plausible task.

## Antimicrobial Lipids

Antimicrobial lipids, which include fatty acids and monoglycerides, are defined as single-chain lipid amphiphiles (Yoon et al., 2018). The antimicrobial properties of these compounds have been known since the 1800s after Koch et al. first documented the antibacterial effects of soap, and later observed that fatty acids could inhibit the growth of *Bacillus anthracis* the causative pathogen of anthrax (Thormar, 2010). Since then the antimicrobial properties of fatty acids and monoglycerides have been extensively explored (Kabara and Vrable, 1977; Desbois and Smith, 2010; Desbois, 2012). Antimicrobial lipids are known to act through a variety of mechanisms, such as increased membrane permeability, cell lysis, disruption of electron transport chain, and inhibition of bacterial enzymes (Yoon et al., 2018). While the antimicrobial properties of these compounds have been known for some time their use as anti-biofilm agents or BEAs is far more recent.

**Figure 9:**
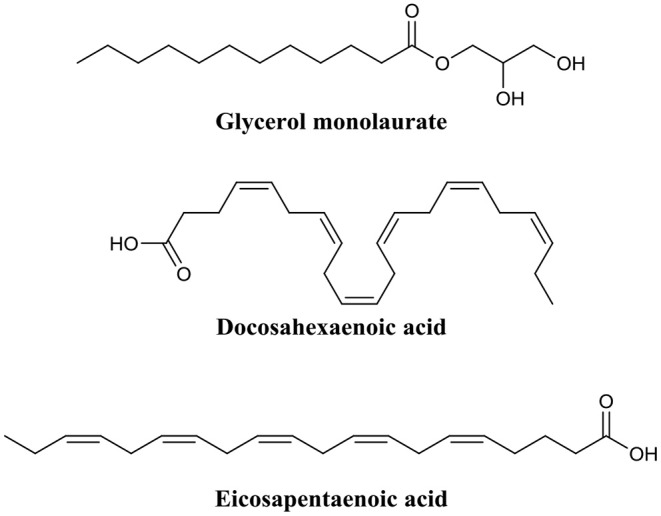
Chemical structures of glycerol monolaurate, docosahexaenoic acid, and eicosapentaenoic acid.

Marshall and Oh were among the first to investigate the use of the monoglyceride, glycerol monolaurate (Figure 9) for the treatment of biofilms (Oh and Marshall, 1995). They examined the biofilm eradication potential of glycerol monolaurate and heat on the foodborne pathogen *Listeria monocytogenes*. Glycerol monolaurate (182 μM) combined with heat (65°C) were found to complete eradicate 7-days-old adherent cells (biofilms) with only 5 min of contact time (Oh and Marshall, 1995). In a subsequent publication by Peterson and Schlievert, glycerol monolaurate alone was found to completely eradicate *S. aureus* and *Haemophilus influenzae* biofilms at a concentration of 1,822 μM (Schlievert and Peterson, 2012). Recently, Santos et al. developed a glycerol monolaurate nanocapsule that was reduced *P. aeruginosa* biofilm biomass by up to 78% when administered at a concentration of 228 μM. These studies clearly evidence that glycerol monolaurate has some level of biofilm eradication potential. However, the active concentration required for biofilm eradication is still quite high compared to other BEAs, particularly for some pathogens. Nevertheless, results using glycerol monolaurate certainly suggest that monoglycerides may someday find use as BEAs.

Shu et al. recently explored the biofilm eradication capabilities of the two fatty acids, docosahexaenoic acid and eicosapentaenoic acid (Figure 9) against *Porphyromonas gingivalis* and *Fusobacterium nucleatum* biofilms (Sun et al., 2016). Administration of docosahexaenoic acid or eicosapentaenoic acid (100 μM) to mature *P. gingivalis* biofilms eradicated a significant proportion of the live cell population (61 and 47%, respectively). The same effect was also evident, albeit to a lower degree, for *F. nucleatum* biofilms (19 and 32%, respectively) (Sun et al., 2016). In a follow-up publication by the same group, these same two fatty acids were assessed for activity against *Streptococcus mutans* biofilms (Sun et al., 2017). Both docosahexaenoic acid and eicosapentaenoic acid were found to significantly damaged the outer membrane of biofilm residing cells (58.8 and 62.5%, respectively), and consequently reduced biofilm thickness by 19 and 42%, respectively, in *S. mutans* (Sun et al., 2017). Importantly, several studies have demonstrated that both docosahexaenoic acid and eicosapentaenoic acid are relatively non-toxic to human cells at concentrations up to 100 μM (docosahexaenoic acid) and 200 μM (eicosapentaenoic acid) (Peng et al., 2012; Yang et al., 2013; Sun et al., 2016).

Considering the low toxicity and promising anti-biofilm and biofilm eradication activity of antimicrobial lipids, there is little question that their use as BEAs merits further investigation. However, as both monoglycerides and fatty acids are present in typical human diets the potential for frequent exposure to these compounds and the subsequent development of resistance is something that must be considered and investigated.

## Anticancer Drugs Mitomycin C AND Cisplatin

**Figure 10:**
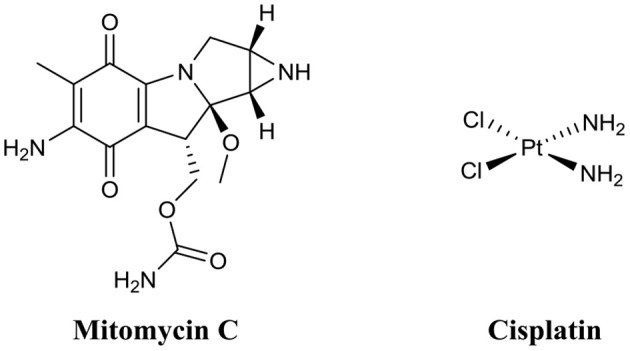
Chemical structures of mitomycin C and cisplatin.

Mitomycin C (Figure 10) is an FDA approved chemotherapy agent with antitumor activity (Doll et al., 1985). It is currently administered for the treatment of a variety of cancers, including cervical, lung, gastric, breast, bladder, head and neck, and pancreatic (Bradner, 2001). Mitomycin C is an amphipathic compound, which enters the cell membrane through passive diffusion (Byfield and Calabro-Jones, 1981) and subsequently initiates DNA crosslinking between adjacent guanine nucleotides (Tomasz, 1995). While the anti-tumor properties of mitomycin C are well-established, its potential as a BEA is only a fairly recent discovery (Kwan et al., 2015). Kwan et al. initially demonstrate that mitomycin C possessed potent planktonic antimicrobial activity against both actively replicating and quiescent (persister) cells against a range of pathogenic bacterial species including *E. coli, S. aureus*, and *P. aeruginosa* (Wood et al., 2013). However, the MICs of mitomycin C against the above species were often higher than that of ciprofloxacin (Wood et al., 2013). Interestingly, when mitomycin C was administered to established biofilms of either *E. coli* O157:H7 or *S. aureus* ATCC 25219 almost complete eradication resulted (<1 × 10^1^ CFU remaining after treatment, >7-log reduction) (Wood et al., 2013). Conversely, under the same conditions ciprofloxacin was significantly less active than mitomycin C against biofilms of *E. coli* O157:H7 (>1 × 10^7^ CFU remaining after treatment) or *S. aureus* ATCC 25219 (>1 × 10^6^ CFU remaining after treatment) (Wood et al., 2013). The biofilm eradication activity of mitomycin C has been attributed to its ability to target and eradicate both actively replicating and persister cells and while encouraging, the concentrations utilized in these experiments (30–40 μM) was significantly higher than the therapeutic concentrations approved for cancer treatment (1.5–6 μM) (Bradner, 2001; Kwan et al., 2015). Thus, the toxicity of these higher concentrations on human health would need to be considered. In an additional study by Wood et al., mitomycin C was also demonstrated to possess potent eradication activity against established *Acinetobacter baumanni* biofilms; however, the concentrations required in this case were even higher (~750 μM) (Cruz-Muniz et al., 2017).

A second anticancer drug with demonstrated biofilm eradication activity is cisplatin (Figure 10) (Chowdhury et al., 2016; Yuan et al., 2018). Cisplatin is also an FDA approved treatment for head and neck, bladder, ovarian, and testicular cancers (Eastman, 1987). Like mitomycin C, cisplatin is also a DNA crosslinker; however, crosslinks occur mostly on the same strand rather than opposing strands like mitomycin C (Eastman, 1987). Wood and coworkers were the first to document the potent eradication activity of cisplatin against established *P. aeruginosa* biofilms (<1 × 10^1^ CFU remaining after treatment, >7-log reduction), however, the dose required for this activity was quite high (833 μM) (Chowdhury et al., 2016). In a subsequent study by Nielsen and colleagues, cisplatin was also shown to have potent *P. aeruginosa* biofilm eradication activity (>1-log reduction) (Yuan et al., 2018). However, complete eradication of biofilms was not observed at the maximum concentration tested (42 μM).

While the biofilm eradication activity of anticancer drugs, such as mitomycin C and cisplatin, might be encouraging, strong consideration must be given to their clinical toxicity. Mitomycin C is known to cause bone marrow damage, lung fibrosis, renal failure, and haemolytic anemia (Doll et al., 1985). Cisplatin can cause bone marrow suppression, kidney damage, hearing impairment, and heart disease (Oun et al., 2018). Interestingly, mitomycin C has also been investigated as a topical treatment for extensive, recurrent conjunctival-corneal squamous cell carcinoma (Shields et al., 2002). In this study, Shields et al. found that mitomycin C was not only highly effective as a topical treatment, but also safe at concentrations up 2.6 mM (Shields et al., 2002). Thus, while anticancer drugs, such as mitomycin C may be too toxic for the treatment of internal biofilm-related infections, they may find use in the treatment of external biofilm-related infections, such as those seen in chronic wounds, diabetic foot ulcers or in skin burns. Furthermore, as both drugs are already FDA-approved and have been in clinical use, they are certainly worth considering as last-resort treatment options for biofilm infections highly recalcintrant to antibiotic therapy.

## Phenazines AND Quinolines

Phenazines are redox-active secondary metabolites, which are produced naturally by many Gram-negative and Gram-positive bacterial species for example, *P. aeruginosa* (Cezairliyan et al., 2013), *Streptomyces* spp. (Karnetova et al., 1983), and *Pantoea agglomerans* (Ali et al., 2016). They consist of a dibenzo annulated pyrazine, and the most well-known example (pyocyanin) originates from *P. aeruginosa* (Lau et al., 2004). Phenazines and their derivatives exhibit activity against both Gram-negative and Gram-positive species; however, Gram-positive species appear to be more susceptible (Baron and Rowe, 1981). Interest in the potential use of phenazines as BEAs arose from the finding that pyocyanin allowed *P. aeruginosa* biofilm infections to outcompete *S. aureus* biofilm infections in the lungs of cystic fibrosis patients (Saiman, 2004; Dietrich et al., 2013).

**Figure 11:**
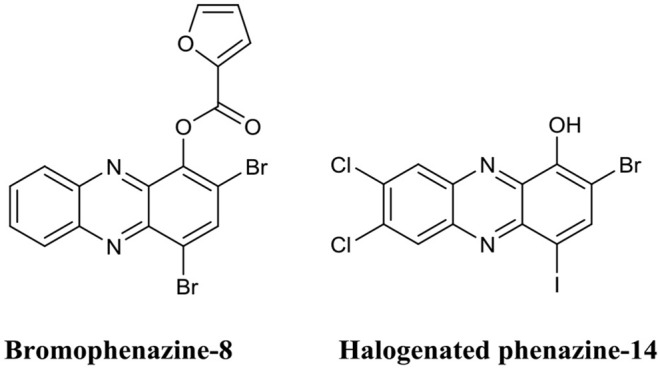
Chemical structures of bromopheazine-8 and halogenated phenazine-14.

Huigens et al. were the first to investigate the biofilm eradication activity of phenazine based compounds and demonstrate their impressive biofilm eradication activity against *S. aureus* biofilms (Garrison et al., 2015b). The most potent of these derivatives was bromophenazine-8 (Figure 11) which completely eradicated biofilms at concentrations between 62.5 and 100 μM. In a subsequent publication by the same group, they prepared an additional library of halogenated phenazines, which this time exhibited biofilm eradication activity against several Gram-positive species (*S. aureus, Staphylococcus epidermidis* and *Enterococcus faecium*) (Garrison et al., 2015a). Halogenated phenazine-14 (Figure 11) exhibited the most potent biofilm eradication activity against all three pathogens with complete eradication occurring at concentrations between 0.2 and 12 μM (Garrison et al., 2015a). Furthermore, the authors also demonstrated that halogenated phenazines are non-toxic to mammalian cells indicating that these compounds or their derivatives represent promising therapeutic candidates for the treatment of Gram-positive biofilm-related infections (Garrison et al., 2015a). The biofilm eradication activity of halogenated quinolones is impressive, at least against Gram-positive pathogens. However, considering the origins of the core phenazine structure (Gram-negative bacterial species), it is doubtful that the activity of this class of BEA will ever extent to Gram-negative pathogens, such as *P. aeruginosa*. Furthermore, bacterially derived phenazines, for example, pyocyanin from *P. aeruginosa*, are well-established virulence factors and key quorum sensing molecules (Lau et al., 2004; Karatuna and Yagci, 2010). Thus, it would also be important to investigate the response of Gram-negative species to these BEAs to ensure that halogenated phenazines do not trigger biofilm formation or increased virulence in bacterial species known to utilize these molecules for quorum sensing. This would be of particular clinical importance in cases where mixed biofilms are typically observed, such as oral and skin infections (Elias and Banin, 2012).

**Figure 12:**
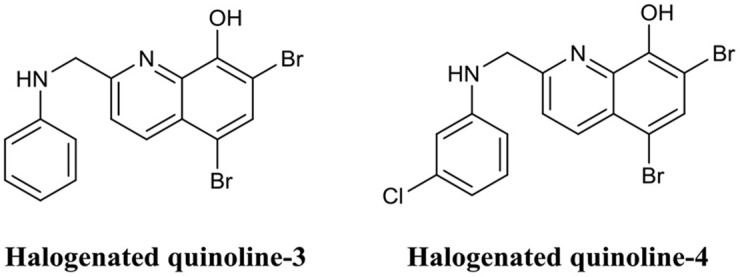
Chemical structures of halogenated quinoline-3 and halogenated quinoline-4.

Quinolines are heterocyclic aromatic compounds which bear some structural resemblance to phenazines. However, unlike phenazines, quinolines are generally associated with antimalarial drugs (Foley and Tilley, 1998). Interestingly, the structural similarities between these two compounds have led to quinolines being investigated as BEAs. Huigens et al. utilized a scaffold hopping strategy (Sun et al., 2012) to develop quinolines based on the halogenated phenazine-14 core structure (Abouelhassan et al., 2014). They produced a variety of halogenated quinolines which exhibited biofilm inhibition activity against *S. aureus* and *S. epidermidis* but possessed little biofilm eradication activity. In subsequent studies by the same group, they improved the biofilm eradication activity of halogenated phenazines against *S. epidermidis* and *E. faecium* (Basak et al., 2015, 2016). Of those, halogenated quinoline-3 (Figure 12) completely eradicated *S. epidermidis* biofilms at only 3.0 μM, while halogenated quinoline-4 eradicated (Figure 12) *E. faecium* biofilm at just 1.0 μM (Basak et al., 2015, 2016). The potential of phenazines as biofilm eradication agents was comprehensively reviewed by the same authors recently (Huigens et al., 2019).

Both halogenated phenazines and quinolines have certainly demonstrated potent biofilm eradication activity. However, their activity appears to be limited to the Gram-positive pathogens *S. aureus, S. epidermidis*, and *E. faecium*. Despite the impressive activity and low cytotoxicity of these compounds, no *in vivo* analyses have been conducted to date. Nevertheless, these compounds are some of the most promising BEAs documented thus far.

## Nitric Oxide-Releasing Antibiotics

**Figure 13:**
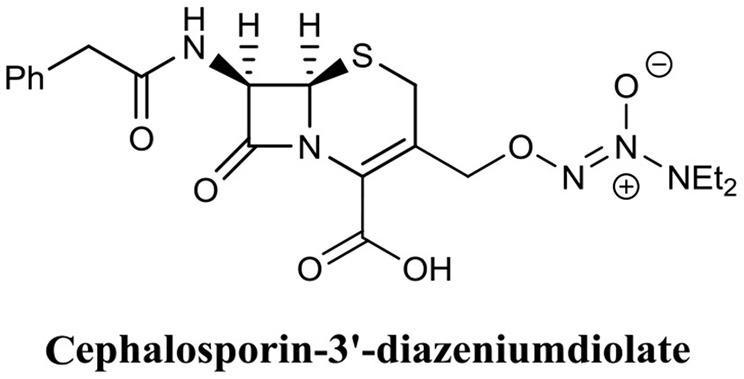
Chemical structure of cephalosporin-3′-diazeniumdiolate.

The use of nitric oxide in biofilm dispersal has been well-documented (Barraud et al., 2006, 2015), however, nitric oxide is a notoriously challenging molecule to handle, and thus its administration and delivery to a target site is often difficult. Furthermore, nitric oxide induces biofilm dispersal at specific concentrations that are sub-lethal to bacteria (below MIC), which means treatment with nitric oxide will require subsequent or combinational treatment with an antimicrobial agent to eradicate dispersed cells. To address the issues surrounding the use of nitric oxide, an innovative approach has been to develop antimicrobials which release nitric oxide or a nitric oxide donor upon interaction with the target site. Kelso and team were able to produce a nitric oxide-releasing prodrug based on the cephalosporin core structure (Barraud et al., 2012). Here they covalently linked the nitric oxide donor diazeniumdiolate (NONOate) to the 3′ position of Cefaloram to produce cephalosporin-3′-diazeniumdiolate (Figure 13), which upon interaction with the bacterial enzyme β-lactamase released the nitric oxide donor that subsequently decomposes to release nitric oxide. When cephalosporin-3′-diazeniumdiolate was administered to established *P. aeruginosa* biofilms, a significant reduction in biofilm-residing cells (70%) was achieved at a concentration of only 10 μM (Barraud et al., 2012). It is not clear however, if these removed cells were killed or remained viable and thus the potential of cephalosporin-3′-diazeniumdiolate as a BEA remains to be demonstrated. Yet this study clearly demonstrated the use of cephalosporin-3′-diazeniumdiolate as a targeted nitric oxide-releasing agent, and more agents have now been reported by the same group that significantly reduce a biofilm population, however, these compounds remain to be tested for biofilm eradication (Yepuri et al., 2013).

In a similar approach, Schoenfisch et al. functionalised an alkyl chain modified poly(amidoamine) (PAMAM) dendrimer with a nitric oxide donor to produce a nitric oxide-releasing antimicrobial agent (Worley et al., 2015). Their lead compounds exhibited a 6-log reduction against *P. aeruginosa* biofilms and a 4-log reduction against *S. aureus* biofilms (Worley et al., 2015). Furthermore, most nitric oxide functionalised conjugates were found to be significantly more potent (2-fold) than their non-nitric oxide containing parent molecules (Worley et al., 2015), a result which suggests that the ability to release nitric oxide greatly improved the biofilm eradication activity of these conjugates. Interestingly, these compounds appear to exhibit a dual action which incorporates the anti-biofilm activity of nitric oxide with the antimicrobial activity of the alkyl chain modified PAMAM dendrimers. As such they represent an interesting new class of BEAs which are potentially dual-acting. Such dual-acting BEAs, combine the activity of two individual compounds to produce a single compound which is more effective than either of its comprising moieties.

## Nitroxide Functionalised Antibiotics

The use of nitric oxide in the BEAs discussed above is complicated by its requirement for release upon contact with the target site (nitric oxide donors must decompose to release nitric oxide). Thus, an another approach that does not require release from the antimicrobial agent would be to utilize a nitric oxide alternative Recently Fairfull-Smith et al. have utilized this approach in the development of nitroxide functionalised antibiotics as BEAs (Verderosa et al., 2016, 2017, 2019a). Nitroxides are not bound by the same limitations as nitric oxide (such as low stability, high reactivity, and gaseous at room temperature). Thus, nitroxides, which have documented anti-biofilm properties (de la Fuente-Núñez et al., 2013; Boase et al., 2018; Woehlk et al., 2019), do not require a delivery or release system. Consequently, they can be synthetically incorporated or linked to other agents, such as antibiotics, without negatively impacting their anti-biofilm properties.

**Figure 14:**
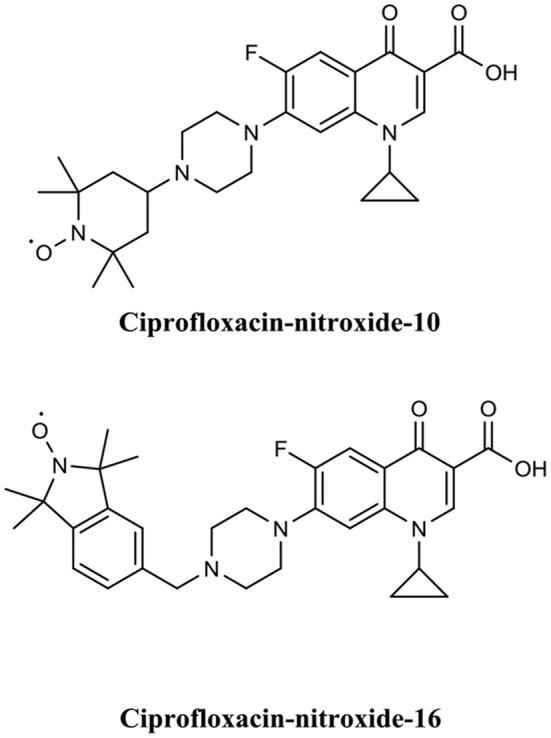
Chemical structures of ciprofloxacin-nitroxide-10 and ciprofloxacin-nitroxide-16.

**Figure 15:**
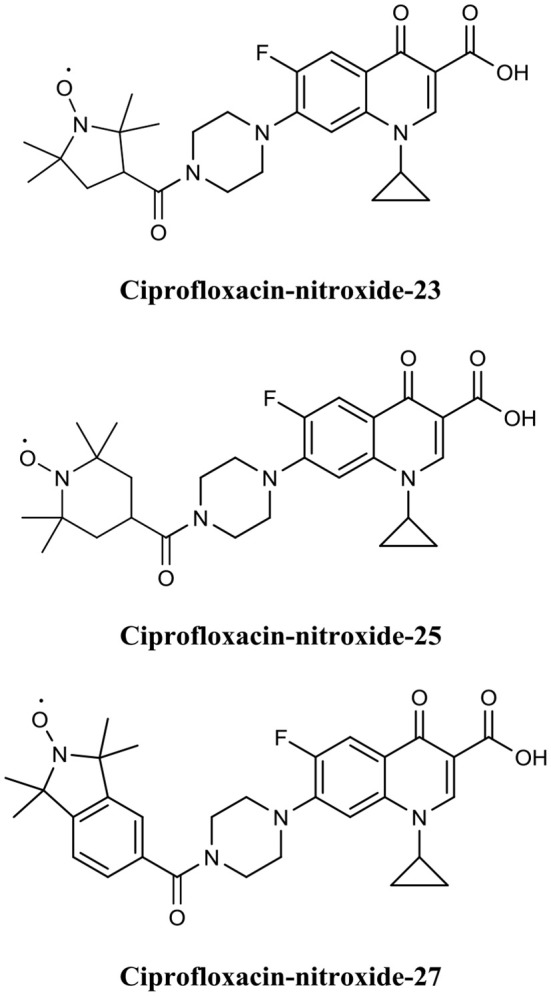
Chemical structures of ciprofloxacin-nitroxide-23, ciprofloxacin-nitroxide-25, and ciprofloxacin-nitroxide-27.

Fairfull-Smith et al. were the first to produce and demonstrate the biofilm eradication activity of nitroxide functionalised antibiotics (Verderosa et al., 2016). Here they synthesized two different ciprofloxacin-nitroxide hybrids (Figure 14) that showed biofilm eradication efficacy against *P. aeruginosa* biofilms (Verderosa et al., 2016). Ciprofloxacin-nitroxide-10 eradicated 95% of biofilm-residing cells at only 40 μM. This represented a major improvement over the parent compound ciprofloxacin, which had little to no effect on biofilm-residing cells in the same assay system (Reffuveille et al., 2014). These studies also evidenced the fundamental role of the free radical nitroxide to the activity of the compound as removal of the free radical character from the hybrid compound significantly reduced its activity as a BEA. In a follow up publication by the same group, a second generation of ciprofloxacin-nitroxide hybrids (Figure 15) were produced and shown to be almost twice as potent as the first-generation hybrids (94% eradication at 20 μM) against *P. aeruginosa* biofilms and also had no mammalian cell toxicity (Verderosa et al., 2017). Recently, Fairfull-Smith et al. produced the third generation of ciprofloxacin-nitroxide hybrids with an optimized nitroxide to antibiotic ratio (Verderosa et al., 2019a). These new hybrids were shown to have improved potency against uropathogenic *E. coli* biofilms (99.7% eradication at 12.5 μM) (Verderosa et al., 2019a). The mechanism of action of these promising BEAs was recently investigated through the development of profluorescent fluoroquinolone nitroxides (Verderosa et al., 2019c). This was the first demonstration that nitroxide-functionalised fluoroquinolones can enter and eradicate both Gram-negative (*P. aeruginosa* and *E. coli*) and Gram-positive pathogen cells (*S. aureus* and *Enterococcus faecalis*) (Verderosa et al., 2019c), demonstrating the broad-spectrum potential of this group of BEAs. In a subsequent publication by Totsika et al., the activity of ciprofloxacin-nitroxides-23, ciprofloxacin-nitroxides-25, and ciprofloxacin-nitroxides-27 (Figure 15) were investigated for efficacy against *S. aureus* biofilms (Verderosa et al., 2019b). Here they found that ciprofloxacin-nitroxide-27 was able to completely (99.9%) eradicate established *S. aureus* biofilm at a concentration of only 64 μM (Verderosa et al., 2019b).

Overall, nitroxide functionalised antibiotics are highly potent (low μM range), exhibit a broad spectrum of activity, have no or low mammalian cytotoxicity, and are based on the structure of a well-established class of antibiotics (fluoroquinolones), making them attractive BEA candidates. In the future, it would be important to examine if other classes of antibiotics can be successfully functionalised with nitroxides to expand this promising group of BEAs.

## Conclusions

Most bacteria in nature exist in the form of biofilms. For the medical profession, biofilms present a considerable challenge, as not only are they associated with most infections in humans, but they are also extremely difficult to treat due to their inherent tolerance to immune responses and antimicrobials. Despite this, most antibiotics are developed and tested against free-living bacteria. Yet our understanding of biofilm formation by several clinically important bacteria and the mechanisms contributing to bacterial antibiotic tolerance has significantly advanced over the past 20 years. This new knowledge has led to the development of several biofilm remediation strategies and the discovery of many promising agents. While the development of anti-biofilm agents that inhibit or disperse biofilms have received significant attention their inherent lack of antimicrobial activity necessitates their use in conjunction with antibiotics, which distances them from offering a clinically reliable standalone solution. The development of BEAs, even though still at early stages, appears capable to address many of these issues with several promising agents already described. The advantage of BEAs is that they do not require supplementation with other drugs and are designed to specifically target biofilm-residing cells. In addition, many BEAs exhibit both anti-biofilm and biofilm eradication activities, such as AMPs and nitroxide functionalised antibiotics. This feature, coupled with their low human cell toxicity, positions BEAs as a potentially complete strategy for the treatment of both planktonic and biofilm-related infections. The BEAs presented in this review exemplify how increasing understanding of biofilm antibiotic tolerance mechanisms can lead to the design and development of new antibiotics that could offer effective solutions against biofilms. As our understanding of these mechanisms continues to improve, so will our ability to develop compounds which can circumvent them. In the near future, research will hopefully unravel the complete mechanisms of biofilm antimicrobial tolerance, and the questions of how to best design and develop new antibiotics will become apparent.

## Author Contributions

AV, MT, and KF-S conceived the concept of the review. AV drafted the manuscript, searched for updated bibliography, and prepared the figures. AV, KF-S, and MT revised, corrected, and edited the manuscript.

### Conflict of Interest

The authors declare that the research was conducted in the absence of any commercial or financial relationships that could be construed as a potential conflict of interest.

## References

[B1] Abouelhassan Y. Garrison A. T. Burch G. M. Wong W. Norwood V. M. Huigens R. W. III. (2014). Discovery of quinoline small molecules with potent dispersal activity against methicillin-resistant *Staphylococcus aureus* and *Staphylococcus epidermidis* biofilms using a scaffold hopping strategy. Bioorg. Med. Chem. Lett. 24, 5076–5080. 10.1016/j.bmcl.2014.09.009 25264073

[B2] Ali H. M. El-Shikh M. S. Salem M. Z. M M. (2016). Isolation of bioactive phenazine-1-carboxamide from the soil bacterium *Pantoea agglomerans* and study of its anticancer potency on different cancer cell lines. J. AOAC Int. 99, 1233–1239. 10.5740/jaoacint.16-0090 27349444

[B3] Allen N. E. (2010). From vancomycin to oritavancin: the discovery and development of a novel lipoglycopeptide antibiotic. Antiinfect. Agents Med. Chem. 9, 23–47. 10.2174/187152110790886745

[B4] Anderl J. N. Franklin M. J. Stewart P. S. (2000). Role of antibiotic penetration limitation in *Klebsiella pneumoniae* biofilm resistance to ampicillin and ciprofloxacin. Antimicrob. Agents Chemother. 44, 1818–1824. 10.1128/AAC.44.7.1818-1824.2000 10858336 PMC89967

[B5] Anderl J. N. Zahller J. Roe F. Stewart P. S. (2003). Role of nutrient limitation and stationary-phase existence in *Klebsiella pneumoniae* biofilm resistance to ampicillin and ciprofloxacin. Antimicrob. Agents Chemother. 47, 1251–1256. 10.1128/AAC.47.4.1251-1256.2003 12654654 PMC152508

[B6] Anderson G. G. O'Toole G. A. (2008). Innate and induced resistance mechanisms of bacterial biofilms. Curr. Top. Microbiol. Immunol. 322, 85–105. 10.1007/978-3-540-75418-3_5 18453273

[B7] Annous B. A. Fratamico P. M. Smith J. L. (2009). Quorum sensing in biofilms: why bacteria behave the way they do. J. Food Sci. 74, R24–R37. 10.1111/j.1750-3841.2008.01022.x 19200115

[B8] Arciola C. R. Campoccia D. Speziale P. Montanaro L. Costerton J. W. (2012). Biofilm formation in *Staphylococcus* implant infections. A review of molecular mechanisms and implications for biofilm-resistant materials. Biomaterials 33, 5967–5982. 10.1016/j.biomaterials.2012.05.031 22695065

[B9] Bagge N. Hentzer M. Andersen J. B. Ciofu O. Givskov M. Hoiby N. (2004a). Dynamics and spatial distribution of beta-lactamase expression in *Pseudomonas aeruginosa* biofilms. Antimicrob. Agents Chemother. 48, 1168–1174. 10.1128/AAC.48.4.1168-1174.2004 15047517 PMC375278

[B10] Bagge N. Schuster M. Hentzer M. Ciofu O. Givskov M. Greenberg E. P.. (2004b). *Pseudomonas aeruginosa* biofilms exposed to imipenem exhibit changes in global gene expression and beta-lactamase and alginate production. Antimicrob. Agents Chemother. 48, 1175–1187. 10.1128/AAC.48.4.1175-1187.2004 15047518 PMC375275

[B11] Baltzer S. A. Brown M. H. (2011). Antimicrobial peptides: promising alternatives to conventional antibiotics. J. Mol. Microbiol. Biotechnol. 20, 228–235. 10.1159/000331009 21894027

[B12] Baron S. S. Rowe J. J. (1981). Antibiotic action of pyocyanin. Antimicrob. Agents Chemother. 20, 814–820. 10.1128/AAC.20.6.814 6798928 PMC181804

[B13] Barraud N. Hassett D. J. Hwang S.-H. Rice S. A. Kjelleberg S. Webb J. S. (2006). Involvement of nitric oxide in biofilm dispersal of *Pseudomonas aeruginosa*. J. Bacteriol. 188, 7344–7353. 10.1128/JB.00779-06 17050922 PMC1636254

[B14] Barraud N. Kardak B. G. Yepuri N. R. Howlin R. P. Webb J. S. Faust S. N.. (2012). Cephalosporin-3′-diazeniumdiolates: targeted NO-donor prodrugs for dispersing bacterial biofilms. Angew. Chem. Int. Ed. Engl. 51, 9057–9060. 10.1002/anie.201202414 22890975

[B15] Barraud N. Kelso M. J. Rice S. A. Kjelleberg S. (2015). Nitric oxide: a key mediator of biofilm dispersal with applications in infectious diseases. Curr. Pharm. Des. 21, 31–42. 10.2174/1381612820666140905112822 25189865

[B16] Barraud N. Storey M. V. Moore Z. P. Webb J. S. Rice S. A. Kjelleberg S. (2009). Nitric oxide-mediated dispersal in single- and multi-species biofilms of clinically and industrially relevant microorganisms. Microb. Biotechnol. 2, 370–378. 10.1111/j.1751-7915.2009.00098.x 21261931 PMC3815757

[B17] Basak A. Abouelhassan Y. Huigens R. W. III. (2015). Halogenated quinolines discovered through reductive amination with potent eradication activities against MRSA, MRSE and VRE biofilms. Org. Biomol. Chem. 13, 10290–10294. 10.1039/C5OB01883H 26414088

[B18] Basak A. Abouelhassan Y. Norwood IV V. M. Bai F. Nguyen M. T. Jin S.. (2016). Synthetically tuning the 2-position of halogenated quinolines: optimizing antibacterial and biofilm eradication activities via alkylation and reductive amination pathways. Chem. Eur. J. 22, 9181–9189. 10.1002/chem.201600926 27245927

[B19] Bazaka K. Jacob M. V. Crawford R. J. Ivanova E. P. (2012). Efficient surface modification of biomaterial to prevent biofilm formation and the attachment of microorganisms. Appl. Microbiol. Biotechnol. 95, 299–311. 10.1007/s00253-012-4144-7 22618687

[B20] Bechinger B. Gorr S. U. (2017). Antimicrobial peptides: mechanisms of action and resistance. J. Dent. Res. 96, 254–260. 10.1177/0022034516679973 27872334 PMC5298395

[B21] Beckloff N. Laube D. Castro T. Furgang D. Park S. Perlin D.. (2007). Activity of an antimicrobial peptide mimetic against planktonic and biofilm cultures of oral pathogens. Antimicrob. Agents Chemother. 51:4125. 10.1128/AAC.00208-07 17785509 PMC2151458

[B22] Behlau I. Gilmore M. S. (2008). Microbial biofilms in ophthalmology and infectious disease. Arch. Ophthalmol. 126, 1572–1581. 10.1001/archopht.126.11.1572 19001227

[B23] Belley A. Neesham-Grenon E. McKay G. Arhin F. F. Harris R. Beveridge T.. (2009). Oritavancin kills stationary-phase and biofilm *Staphylococcus aureus* cells *in vitro*. Antimicrob. Agents Chemother. 53:918. 10.1128/AAC.00766-08 19104027 PMC2650528

[B24] Beloin C. Roux A. Ghigo J. M. (2008). *Escherichia coli* biofilms. Curr. Top. Microbiol. 322, 249–289. 10.1007/978-3-540-75418-3_12 18453280 PMC2864707

[B25] Bigger J. (1944). Treatment of *Staphylococcal* infections with penicillin by intermittent sterilisation. Lancet 244, 497–500. 10.1016/S0140-6736(00)74210-3

[B26] Boase N. R. B. Torres M. D. T. Fletcher N. L. de la Fuente-Nunez C. Fairfull-Smith K. E. (2018). Polynitroxide copolymers to reduce biofilm fouling on surfaces. Polym. Chem. 9, 5308–5318. 10.1039/C8PY01101J

[B27] Borriello G. Werner E. Roe F. Kim A. M. Ehrlich G. D. Stewart P. S. (2004). Oxygen limitation contributes to antibiotic tolerance of *Pseudomonas aeruginosa* in biofilms. Antimicrob. Agents Chemother. 48, 2659–2664. 10.1128/AAC.48.7.2659-2664.2004 15215123 PMC434183

[B28] Bradner W. T. (2001). Mitomycin C: a clinical update. Cancer Treat. Rev. 27, 35–50. 10.1053/ctrv.2000.0202 11237776

[B29] Brown K. L. Hancock R. E. (2006). Cationic host defense (antimicrobial) peptides. Curr. Opin. Immunol. 18, 24–30. 10.1016/j.coi.2005.11.004 16337365

[B30] Brown M. R. Allison D. G. Gilbert P. (1988). Resistance of bacterial biofilms to antibiotics a growth-rate related effect? J. Antimicrob. Chemother. 22, 777–780. 10.1093/jac/22.6.777 3072331

[B31] Burton M. F. Steel P. G. (2009). The chemistry and biology of LL-37. Nat. Prod. Rep. 26, 1572–1584. 10.1039/b912533g 19936387

[B32] Byfield J. E. Calabro-Jones P. M. (1981). Carrier-dependent and carrier-independent transport of anti-cancer alkylating agents. Nature 294, 281–283. 10.1038/294281a0 6975441

[B33] Campanac C. Pineau L. Payard A. Baziard-Mouysset G. Roques C. (2002). Interactions between biocide cationic agents and bacterial biofilms. Antimicrob. Agents Chemother. 46, 1469–1474. 10.1128/AAC.46.5.1469-1474.2002 11959584 PMC127133

[B34] Campoccia D. Montanaro L. Arciola C. R. (2013). A review of the biomaterials technologies for infection-resistant surfaces. Biomaterials 34, 8533–8554. 10.1016/j.biomaterials.2013.07.089 23953781

[B35] Cezairliyan B. Vinayavekhin N. Grenfell-Lee D. Yuen G. J. Saghatelian A. Ausubel F. M. (2013). Identification of *Pseudomonas aeruginosa* phenazines that kill *Caenorhabditis elegans*. PLoS Pathog. 9:e1003101. 10.1371/journal.ppat.1003101 23300454 PMC3536714

[B36] Chennupati S. K. Chiu A. G. Tamashiro E. Banks C. A. Cohen M. B. Bleier B. S.. (2009). Effects of an LL-37-derived antimicrobial peptide in an animal model of biofilm *Pseudomonas sinusitis*. Am. J. Rhinol. Allergy 23, 46–51. 10.2500/ajra.2009.23.3261 19379612

[B37] Chowdhury N. Wood T. L. Martinez-Vazquez M. Garcia-Contreras R. Wood T. K. (2016). DNA-crosslinker cisplatin eradicates bacterial persister cells. Biotechnol. Bioeng. 113, 1984–1992. 10.1002/bit.25963 26914280

[B38] Costerton J. W. Cheng K. J. Geesey G. G. Ladd T. I. Nickel J. C. Dasgupta M.. (1987). Bacterial biofilms in nature and disease. Annu. Rev. Microbiol. 41, 435–464. 10.1146/annurev.mi.41.100187.002251 3318676

[B39] Costerton J. W. Stewart P. S. Greenberg E. P. (1999). Bacterial biofilms: a common cause of persistent infections. Science 284, 1318–1322. 10.1126/science.284.5418.1318 10334980

[B40] Cruz-Muniz M. Y. Lopez-Jacome L. E. Hernandez-Duran M. Franco-Cendejas R. Licona-Limon P. Ramos-Balderas J. L.. (2017). Repurposing the anticancer drug mitomycin C for the treatment of persistent *Acinetobacter baumannii* infections. Int. J. Antimicrob. Agents 49, 88–92. 10.1016/j.ijantimicag.2016.08.022 27939675

[B41] Darpo B. Lee S. K. Moon T. E. Sills N. Mason J. W. (2010). Oritavancin, a new lipoglycopeptide antibiotic: results from a thorough QT study. J. Clin. Pharmacol. 50, 895–903. 10.1177/0091270009355449 20484618

[B42] Dashper S. G. Brien-Simpson N. M. Cross K. J. Paolini R. A. Hoffmann B. Catmull D. V.. (2005). Divalent metal cations increase the activity of the antimicrobial peptide kappacin. Antimicrob. Agents Chemother. 49:2322. 10.1128/AAC.49.6.2322-2328.2005 15917528 PMC1140507

[B43] Davenport E. K. Call D. R. Beyenal H. (2014). Differential protection from tobramycin by extracellular polymeric substances from *Acinetobacter baumannii* and *Staphylococcus aureus* biofilms. Antimicrob. Agents Chemother. 58, 4755–4761. 10.1128/AAC.03071-14 24913166 PMC4136036

[B44] Davies D. (2003). Understanding biofilm resistance to antibacterial agents. Nat. Rev. Drug Discov. 2, 114–122. 10.1038/nrd1008 12563302

[B45] de la Fuente-Núñez C. Reffuveille F. Fairfull-Smith K. E. Hancock R. E. W. (2013). Effect of nitroxides on swarming motility and biofilm formation, multicellular behaviors in *Pseudomonas aeruginosa*. Antimicrob. Agents Chemother. 57, 4877–4881. 10.1128/AAC.01381-13 23877682 PMC3811460

[B46] Delcaru C. Alexandru I. Podgoreanu P. Grosu M. Stavropoulos E. Chifiriuc M. C.. (2016). Microbial biofilms in urinary tract infections and prostatitis: etiology, pathogenicity, and combating strategies. Pathogens 5:65. 10.3390/pathogens5040065 27916925 PMC5198165

[B47] Desbois A. P. (2012). Potential applications of antimicrobial fatty acids in medicine, agriculture and other industries. Recent Pat. Antiinfect. Drug Discov. 7, 111–122. 10.2174/157489112801619728 22630821

[B48] Desbois A. P. Smith V. J. (2010). Antibacterial free fatty acids: activities, mechanisms of action and biotechnological potential. Appl. Microbiol. Biotechnol. 85, 1629–1642. 10.1007/s00253-009-2355-3 19956944

[B49] Dickschat J. S. (2010). Quorum sensing and bacterial biofilms. Nat. Prod. Rep. 27, 343–369. 10.1039/b804469b 20179876

[B50] Dietrich L. E. P. Okegbe C. Price-Whelan A. Sakhtah H. Hunter R. C. Newman D. K. (2013). Bacterial community morphogenesis is intimately linked to the intracellular redox state. J. Bacteriol. 195:1371. 10.1128/JB.02273-12 23292774 PMC3624522

[B51] Doll D. C. Weiss R. B. Issell B. F. (1985). Mitomycin: ten years after approval for marketing. J. Clin. Oncol. 3, 276–286. 10.1200/JCO.1985.3.2.276 3881563

[B52] Donlan R. M. Costerton J. W. (2002). Biofilms: survival mechanisms of clinically relevant microorganisms. Clin. Microbiol. Rev. 15, 167–193. 10.1128/CMR.15.2.167-193.2002 11932229 PMC118068

[B53] Dunne W. M. Jr. (2002). Bacterial adhesion: seen any good biofilms lately? Clin. Microbiol. Rev. 15, 155–166. 10.1128/CMR.15.2.155-166.2002 11932228 PMC118072

[B54] Eastman A. (1987). The formation, isolation and characterization of DNA adducts produced by anticancer platinum complexes. Pharmacol. Ther. 34, 155–166. 10.1016/0163-7258(87)90009-X 3317449

[B55] Eckert R. Brady K. M. Greenberg E. P. Qi F. Yarbrough D. K. He J.. (2006). Enhancement of antimicrobial activity against *Pseudomonas aeruginosa* by coadministration of G10KHc and tobramycin. Antimicrob. Agents Chemother. 50:3833. 10.1128/AAC.00509-06 16940063 PMC1635211

[B56] Elias S. Banin E. (2012). Multi-species biofilms: living with friendly neighbors. FEMS Microbiol. Rev. 36, 990–1004. 10.1111/j.1574-6976.2012.00325.x 22229800

[B57] Farrell D. J. Robbins M. Rhys-Williams W. Love W. G. (2010). *In vitro* activity of XF-73, a novel antibacterial agent, against antibiotic-sensitive and -resistant Gram-positive and Gram-negative bacterial species. Int. J. Antimicrob. Agents 35, 531–536. 10.1016/j.ijantimicag.2010.02.008 20346634

[B58] Farrell D. J. Robbins M. Rhys-Williams W. Love W. G. (2011). Investigation of the potential for mutational resistance to XF-73, retapamulin, mupirocin, fusidic acid, daptomycin, and vancomycin in methicillin-resistant *Staphylococcus aureus* isolates during a 55-passage study. Antimicrob. Agents Chemother. 55:1177. 10.1128/AAC.01285-10 21149626 PMC3067113

[B59] Field T. R. White A. Elborn J. S. Tunney M. M. (2005). Effect of oxygen limitation on the *in vitro* antimicrobial susceptibility of clinical isolates of *Pseudomonas aeruginosa* grown planktonically and as biofilms. Eur. J. Clin. Microbiol. Infect. Dis. 24, 677–687. 10.1007/s10096-005-0031-9 16249934

[B60] Fleming D. Rumbaugh K. (2018). The consequences of biofilm dispersal on the host. Sci. Rep. 8:10738. 10.1038/s41598-018-29121-2 30013112 PMC6048044

[B61] Fleming D. Rumbaugh K. P. (2017). Approaches to dispersing medical biofilms. Microorganisms 5:15. 10.3390/microorganisms5020015 28368320 PMC5488086

[B62] Flemming H.-C. Neu T. R. Wozniak D. J. (2007). The EPS matrix: the house of biofilm cells. J. Bacteriol. 189, 7945–7947. 10.1128/JB.00858-07 17675377 PMC2168682

[B63] Flemming K. Klingenberg C. Cavanagh J. P. Sletteng M. Stensen W. Svendsen J. S.. (2009). High *in vitro* antimicrobial activity of synthetic antimicrobial peptidomimetics against *staphylococcal* biofilms. J. Antimicrob. Chemother. 63, 136–145. 10.1093/jac/dkn464 19010828

[B64] Foley M. Tilley L. (1998). Quinoline antimalarials: mechanisms of action and resistance and prospects for new agents. Pharmacol. Ther. 79, 55–87. 10.1016/S0163-7258(98)00012-6 9719345

[B65] Forman M. E. Jennings M. C. Wuest W. M. Minbiole K. P. (2016). Building a better quaternary ammonium compound (QAC): branched tetracationic antiseptic amphiphiles. Chem. Med. Chem. 11, 1401–1405. 10.1002/cmdc.201600176 27245743

[B66] Francolini I. Donelli G. (2010). Prevention and control of biofilm-based medical-device-related infections. FEMS Immunol. Med. Microbiol. 59, 227–238. 10.1111/j.1574-695X.2010.00665.x 20412300

[B67] Garrison A. T. Abouelhassan Y. Kallifidas D. Bai F. Ukhanova M. Mai V.. (2015a). Halogenated phenazines that potently eradicate biofilms, MRSA persister cells in non-biofilm cultures, and *Mycobacterium tuberculosis*. Angew. Chem. Int. Ed. Engl. 54, 14819–14823. 10.1002/anie.201508155 26480852

[B68] Garrison A. T. Bai F. Abouelhassan Y. Paciaroni N. G. Jin S. Huigens R. W. III (2015b). Bromophenazine derivatives with potent inhibition, dispersion and eradication activities against *Staphylococcus aureus* biofilms. RSC Adv. 5, 1120–1124. 10.1039/C4RA08728C

[B69] Gellatly S. L. Hancock R. E. (2013). *Pseudomonas aeruginosa*: new insights into pathogenesis and host defenses. Pathog. Dis. 67, 159–173. 10.1111/2049-632X.12033 23620179

[B70] Gordon R. J. Lowy F. D. (2008). Pathogenesis of methicillin-resistant *Staphylococcus aureus* infection. Clin. Infect. Dis. 46, S350–S359. 10.1086/533591 18462090 PMC2474459

[B71] Gordon Y. J. Huang L. C. Romanowski E. G. Yates K. A. Proske R. J. McDermott A. M. (2005). Human cathelicidin (LL-37), a multifunctional peptide, is expressed by ocular surface epithelia and has potent antibacterial and antiviral activity. Curr. Eye Res. 30, 385–394. 10.1080/02713680590934111 16020269 PMC1497871

[B72] Guilhen C. Forestier C. Balestrino D. (2017). Biofilm dispersal: multiple elaborate strategies for dissemination of bacteria with unique properties. Mol. Microbiol. 105, 188–210. 10.1111/mmi.13698 28422332

[B73] Habash M. Reid G. (1999). Microbial biofilms: their development and significance for medical device-related infections. J. Clin. Pharmacol. 39, 887–898. 10.1177/00912709922008506 10471979

[B74] Hannan S. Ready D. Jasni A. S. Rogers M. Pratten J. Roberts A. P. (2010). Transfer of antibiotic resistance by transformation with eDNA within oral biofilms. FEMS Immunol. Med. Microbiol. 59, 345–349. 10.1111/j.1574-695X.2010.00661.x 20337719

[B75] Harris F. Dennison S. R. Phoenix D. A. (2009). Anionic antimicrobial peptides from eukaryotic organisms. Curr. Protein Pept. Sci. 10, 585–606. 10.2174/138920309789630589 19751192

[B76] Harrison J. J. Ceri H. Roper N. J. Badry E. A. Sproule K. M. Turner R. J. (2005a). Persister cells mediate tolerance to metal oxyanions in *Escherichia coli*. Microbiology 151, 3181–3195. 10.1099/mic.0.27794-0 16207903

[B77] Harrison J. J. Turner R. J. Ceri H. (2005b). Persister cells, the biofilm matrix and tolerance to metal cations in biofilm and planktonic *Pseudomonas aeruginosa*. Environ. Microbiol. 7, 981–994. 10.1111/j.1462-2920.2005.00777.x 15946294

[B78] Hausner M. Wuertz S. (1999). High rates of conjugation in bacterial biofilms as determined by quantitative *in situ* analysis. Appl. Environ. Microbiol. 65, 3710–3713. 10427070 10.1128/aem.65.8.3710-3713.1999 PMC91555

[B79] Hell E. Giske C. G. Nelson A. Romling U. Marchini G. (2010). Human cathelicidin peptide LL37 inhibits both attachment capability and biofilm formation of *Staphylococcus epidermidis*. Lett. Appl. Microbiol. 50, 211–215. 10.1111/j.1472-765X.2009.02778.x 20002576

[B80] Hobby G. L. Meyer K. Chaffee E. (1942). Observations on the mechanism of action of penicillin. Proc. Soc. Exp. Biol. Med. 50, 281–285. 10.3181/00379727-50-13773

[B81] Hoiby N. Bjarnsholt T. Givskov M. Molin S. Ciofu O. (2010). Antibiotic resistance of bacterial biofilms. Int. J. Antimicrob. Agents 35, 322–332. 10.1016/j.ijantimicag.2009.12.011 20149602

[B82] Hou S. Liu Z. Young A. W. Mark S. L. Kallenbach N. R. Ren D. (2010). Effects of Trp- and Arg-containing antimicrobial-peptide structure on inhibition of *Escherichia coli* planktonic growth and biofilm formation. Appl. Environ. Microbiol. 76, 1967–1974. 10.1128/AEM.02321-09 20097816 PMC2838007

[B83] Hou S. Zhou C. Liu Z. Young A. W. Shi Z. Ren D.. (2009). Antimicrobial dendrimer active against *Escherichia coli* biofilms. Bioorg. Med. Chem. Lett. 19, 5478–5481. 10.1016/j.bmcl.2009.07.077 19682902

[B84] Huigens R. W. III Abouelhassan Y. Yang H. (2019). Phenazine antibiotic inspired discovery of bacterial biofilm-eradicating agents. Chem. Med. Chem. 20, 1–19. 10.1002/cbic.201900116 PMC7325843 30811834

[B85] Ioannou C. J. Hanlon G. W. Denyer S. P. (2007). Action of disinfectant quaternary ammonium compounds against *Staphylococcus aureus*. Antimicrob. Agents Chemother. 51, 296–306. 10.1128/AAC.00375-06 17060529 PMC1797692

[B86] James G. A. Swogger E. Wolcott R. Pulcini E. D. Secor P. Sestrich J.. (2008). Biofilms in chronic wounds. Wound Repair Regen. 16, 37–44. 10.1111/j.1524-475X.2007.00321.x 18086294

[B87] Jennings M. C. Ator L. E. Paniak T. J. Minbiole K. P. Wuest W. M. (2014). Biofilm-eradicating properties of quaternary ammonium amphiphiles: simple mimics of antimicrobial peptides. Chem. Bio. Chem. 15, 2211–2215. 10.1002/cbic.201402254 25147134

[B88] Kabara J. J. Vrable R. (1977). Antimicrobial lipids: natural and synthetic fatty acids and monoglycerides. Lipids 12, 753–759. 10.1007/BF02570908 409896

[B89] Kang J. Dietz M. J. Li B. (2019). Antimicrobial peptide LL-37 is bactericidal against *Staphylococcus aureus* biofilms. PLoS ONE 14:e0216676. 10.1371/journal.pone.0216676 31170191 PMC6553709

[B90] Karatuna O. Yagci A. (2010). Analysis of quorum sensing-dependent virulence factor production and its relationship with antimicrobial susceptibility in *Pseudomonas aeruginosa* respiratory isolates. Clin. Microbiol. Infect. 16, 1770–1775. 10.1111/j.1469-0691.2010.03177.x 20132256

[B91] Karnetova J. Tax J. Stajner K. Vanek Z. Krumphanzl V. (1983). Production of phenazines by *Streptomyces cinnamonensis*. Folia Microbiol. (Praha) 28, 51–53. 10.1007/BF02877385 6832658

[B92] Katsikogianni M. Missirlis Y. F. (2004). Concise review of mechanisms of bacterial adhesion to biomaterials and of techniques used in estimating bacteria-material interactions. Eur. Cell Mater. 8, 37–57. 10.22203/eCM.v008a05 15593018

[B93] Keren I. Kaldalu N. Spoering A. Wang Y. Lewis K. (2004a). Persister cells and tolerance to antimicrobials. FEMS Microbiol. Lett. 230, 13–18. 10.1016/S0378-1097(03)00856-5 14734160

[B94] Keren I. Shah D. Spoering A. Kaldalu N. Lewis K. (2004b). Specialized persister cells and the mechanism of multidrug tolerance in *Escherichia coli*. J. Bacteriol. 186, 8172–8180. 10.1128/JB.186.24.8172-8180.2004 15576765 PMC532439

[B95] Kwan B. W. Chowdhury N. Wood T. K. (2015). Combatting bacterial infections by killing persister cells with mitomycin C. Environ. Microbiol. 17, 4406–4414. 10.1111/1462-2920.12873 25858802

[B96] Lau G. W. Hassett D. J. Ran H. Kong F. (2004). The role of pyocyanin in *Pseudomonas aeruginosa* infection. Trends Mol. Med. 10, 599–606. 10.1016/j.molmed.2004.10.002 15567330

[B97] Lebeaux D. Ghigo J.-M. Beloin C. (2014). Biofilm-related infections: bridging the gap between clinical management and fundamental aspects of recalcitrance toward antibiotics. Microbiol. Mol. Biol. Rev. 78, 510–543. 10.1128/MMBR.00013-14 25184564 PMC4187679

[B98] Lewis K. (2001). Riddle of biofilm resistance. Antimicrob. Agents Chemother. 45, 999–1007. 10.1128/AAC.45.4.999-1007.2001 11257008 PMC90417

[B99] Lewis K. (2005). Persister cells and the riddle of biofilm survival. Biochemistry (Mosc). 70, 267–274. 10.1007/s10541-005-0111-6 15807669

[B100] Lewis K. (2010). Persister cells. Annu. Rev. Microbiol. 64, 357–372. 10.1146/annurev.micro.112408.134306 20528688

[B101] Luppens S. B. I. Reij M. W. van der Heijden R. W. L. Rombouts F. M. Abee T. (2002). Development of a standard test to assess the resistance of *Staphylococcus aureus* biofilm cells to disinfectants. Appl. Environ. Microbiol. 68, 4194–4200. 10.1128/AEM.68.9.4194-4200.2002 12200265 PMC124130

[B102] Machado D. Castro J. Palmeira-de-Oliveira A. Martinez-de-Oliveira J. Cerca N. (2016). Bacterial vaginosis biofilms: challenges to current therapies and emerging solutions. Front. Microbiol. 6:1528. 10.3389/fmicb.2015.01528 26834706 PMC4718981

[B103] Marvasi M. Chen C. Carrazana M. Durie I. A. Teplitski M. (2014). Systematic analysis of the ability of nitric oxide donors to dislodge biofilms formed by *Salmonella enterica* and *Escherichia coli* O157:H7. AMB Express 4, 1–11. 10.1186/s13568-014-0042-y 24995149 PMC4070026

[B104] McDougald D. Rice S. A. Barraud N. Steinberg P. D. Kjelleberg S. (2012). Should we stay or should we go: mechanisms and ecological consequences for biofilm dispersal. Nat. Rev. Microbiol. 10, 39–50. 10.1038/nrmicro2695 22120588

[B105] Munita J. M. Arias C. A. (2016). Mechanisms of antibiotic resistance. Microbiol. Spectr. 4:10.1128/microbiolspec.VMBF-0016-2015. 10.1128/microbiolspec.VMBF-0016-2015 27227291 PMC4888801

[B106] Nablo B. J. Rothrock A. R. Schoenfisch M. H. (2005). Nitric oxide-releasing sol–gels as antibacterial coatings for orthopedic implants. Biomaterials 26, 917–924. 10.1016/j.biomaterials.2004.03.031 15353203

[B107] Nadell C. D. Xavier J. B. Levin S. A. Foster K. R. (2008). The evolution of quorum sensing in bacterial biofilms. PLoS Biol. 6:e14. 10.1371/journal.pbio.0060014 18232735 PMC2214811

[B108] Nijnik A. Hancock R. E. (2009). The roles of cathelicidin LL-37 in immune defences and novel clinical applications. Curr. Opin. Hematol. 16, 41–47. 10.1097/MOH.0b013e32831ac517 19068548

[B109] Oh D.-H. Marshall D. L. (1995). Destruction of *Listeria monocytogenes* biofilms on stainless steel using monolaurin and heat. J. Food Prot. 58, 251–255. 10.4315/0362-028X-58.3.251 31137284

[B110] Omar A. Wright J. B. Schultz G. Burrell R. Nadworny P. (2017). Microbial biofilms and chronic wounds. Microorganisms 5:9. 10.3390/microorganisms5010009 28272369 PMC5374386

[B111] Ooi N. Miller K. Randall C. Rhys-Williams W. Love W. Chopra I. (2010). XF-70 and XF-73, novel antibacterial agents active against slow-growing and non-dividing cultures of *Staphylococcus aureus* including biofilms. J. Antimicrob. Chemother. 65, 72–78. 10.1093/jac/dkp409 19889790

[B112] Oun R. Moussa Y. E. Wheate N. J. (2018). The side effects of platinum-based chemotherapy drugs: a review for chemists. Dalton Trans. 47, 6645–6653. 10.1039/C8DT00838H 29632935

[B113] Overhage J. Campisano A. Bains M. Torfs E. C. Rehm B. H. Hancock R. E. (2008). Human host defense peptide LL-37 prevents bacterial biofilm formation. Infect. Immun. 76, 4176–4182. 10.1128/IAI.00318-08 18591225 PMC2519444

[B114] Penesyan A. Gillings M. Paulsen I. T. (2015). Antibiotic discovery: combatting bacterial resistance in cells and in biofilm communities. Molecules 20, 5286–5298. 10.3390/molecules20045286 25812150 PMC6272253

[B115] Peng Y. Zheng Y. Zhang Y. Zhao J. Chang F. Lu T.. (2012). Different effects of omega-3 fatty acids on the cell cycle in C2C12 myoblast proliferation. Mol. Cell. Biochem. 367, 165–173. 10.1007/s11010-012-1329-4 22610825

[B116] Post J. C. (2001). Direct evidence of bacterial biofilms in otitis media. Laryngoscope 111, 2083–2094. 10.1097/00005537-200112000-00001 11802002

[B117] Rabin N. Zheng Y. Opoku-Temeng C. Du Y. Bonsu E. Sintim H. O. (2015). Agents that inhibit bacterial biofilm formation. Fut. Med. Chem. 7, 647–671. 10.4155/fmc.15.7 25921403

[B118] Rachid S. Ohlsen K. Witte W. Hacker J. Ziebuhr W. (2000). Effect of subinhibitory antibiotic concentrations on polysaccharide intercellular adhesin expression in biofilm-forming *Staphylococcus epidermidis*. Antimicrob. Agents Chemother. 44, 3357–3363. 10.1128/AAC.44.12.3357-3363.2000 11083640 PMC90205

[B119] Redelman C. V. Chakravarty S. Anderson G. G. (2014). Antibiotic treatment of *Pseudomonas aeruginosa* biofilms stimulates expression of the magnesium transporter gene mgtE. Microbiology 160, 165–178. 10.1099/mic.0.070144-0 24162608

[B120] Reffuveille F. de la Fuente-Núñez C. Hancock R. E. W. Fairfull-Smith K. E. (2015). Potentiation of ciprofloxacin action against gram-negative bacterial biofilms by a nitroxide. Pathog. Dis. 73:ftv016. 10.1093/femspd/ftv016 25736816

[B121] Reffuveille F. de la Fuente-Nunez C. Mansour S. Hancock R. E. (2014). A broad-spectrum antibiofilm peptide enhances antibiotic action against bacterial biofilms. Antimicrob. Agents Chemother. 58, 5363–5371. 10.1128/AAC.03163-14 24982074 PMC4135845

[B122] Roizman D. Vidaillac C. Givskov M. Yang L. (2017). *In vitro* evaluation of biofilm dispersal as a therapeutic strategy to restore antimicrobial efficacy. Antimicrob. Agents Chemother. 61, e01088–e01017. 10.1128/AAC.01088-17 28760898 PMC5610510

[B123] Roy R. Tiwari M. Donelli G. Tiwari V. (2018). Strategies for combating bacterial biofilms: a focus on anti-biofilm agents and their mechanisms of action. Virulence 9, 522–554. 10.1080/21505594.2017.1313372 28362216 PMC5955472

[B124] Russell A. D. (2003). Biocide use and antibiotic resistance: the relevance of laboratory findings to clinical and environmental situations. Lancet Infect. Dis. 3, 794–803. 10.1016/S1473-3099(03)00833-8 14652205

[B125] Rybak M. J. McGrath B. J. (1996). Combination antimicrobial therapy for bacterial infections. Guidelines for the clinician. Drugs 52, 390–405. 10.2165/00003495-199652030-00005 8875129

[B126] Sailer F. C. Meberg B. M. Young K. D. (2003). beta-Lactam induction of colanic acid gene expression in *Escherichia coli*. FEMS Microbiol. Lett. 226, 245–249. 10.1016/S0378-1097(03)00616-5 14553918

[B127] Saiman L. (2004). Microbiology of early CF lung disease. Paediatr. Respir Rev. 5, S367–S369. 10.1016/S1526-0542(04)90065-6 14980298

[B128] Schachter B. (2003). Slimy business-the biotechnology of biofilms. Nat. Biotechnol. 21, 361–365. 10.1038/nbt0403-361 12665817

[B129] Schlievert P. M. Peterson M. L. (2012). Glycerol monolaurate antibacterial activity in broth and biofilm cultures. PLoS ONE 7:e40350. 10.1371/journal.pone.0040350 22808139 PMC3394780

[B130] Shai Y. (1999). Mechanism of the binding, insertion and destabilization of phospholipid bilayer membranes by alpha-helical antimicrobial and cell non-selective membrane-lytic peptides. Biochim. Biophys. Acta 1462, 55–70. 10.1016/S0005-2736(99)00200-X 10590302

[B131] Shields C. L. Naseripour M. Shields J. A. (2002). Topical mitomycin C for extensive, recurrent conjunctival-corneal squamous cell carcinoma. Am. J. Ophthalmol. 133, 601–606. 10.1016/S0002-9394(02)01400-9 11992855

[B132] Simões M. Simões L. C. Vieira M. J. (2010). A review of current and emergent biofilm control strategies. LWT Food Sci. Technol. 43, 573–583. 10.1016/j.lwt.2009.12.008

[B133] Spoering A. L. Lewis K. (2001). Biofilms and planktonic cells of *Pseudomonas aeruginosa* have similar resistance to killing by antimicrobials. J. Bacteriol. 183, 6746–6751. 10.1128/JB.183.23.6746-6751.2001 11698361 PMC95513

[B134] Spoering A. L. Vulić M. Lewis K. (2006). GlpD and PlsB participate in persister cell formation in *Escherichia coli*. J. Bacteriol. 188:5136. 10.1128/JB.00369-06 16816185 PMC1539972

[B135] Stewart P. S. (2002). Mechanisms of antibiotic resistance in bacterial biofilms. Int. J Med. Microbiol. 292, 107–113. 10.1078/1438-4221-00196 12195733

[B136] Stewart P. S. William Costerton J. (2001). Antibiotic resistance of bacteria in biofilms. Lancet 358, 135–138. 10.1016/S0140-6736(01)05321-1 11463434

[B137] Stone G. Wood P. Dixon L. Keyhan M. Matin A. (2002). Tetracycline rapidly reaches all the constituent cells of uropathogenic *Escherichia coli* biofilms. Antimicrob. Agents Chemother. 46, 2458–2461. 10.1128/AAC.46.8.2458-2461.2002 12121918 PMC127323

[B138] Stoodley P. Sauer K. Davies D. G. Costerton J. W. (2002). Biofilms as complex differentiated communities. Annu. Rev. Microbiol. 56, 187–209. 10.1146/annurev.micro.56.012302.160705 12142477

[B139] Sun H. Tawa G. Wallqvist A. (2012). Classification of scaffold-hopping approaches. Drug Discov. Today 17, 310–324. 10.1016/j.drudis.2011.10.024 22056715 PMC3328312

[B140] Sun M. Dong J. Xia Y. Shu R. (2017). Antibacterial activities of docosahexaenoic acid (DHA) and eicosapentaenoic acid (EPA) against planktonic and biofilm growing *Streptococcus mutans*. Microb. Pathog. 107, 212–218. 10.1016/j.micpath.2017.03.040 28373143

[B141] Sun M. Zhou Z. Dong J. Zhang J. Xia Y. Shu R. (2016). Antibacterial and antibiofilm activities of docosahexaenoic acid (DHA) and eicosapentaenoic acid (EPA) against periodontopathic bacteria. Microb. Pathog. 99, 196–203. 10.1016/j.micpath.2016.08.025 27565090

[B142] Szomolay B. Klapper I. Dockery J. Stewart P. S. (2005). Adaptive responses to antimicrobial agents in biofilms. Environ. Microbiol. 7, 1186–1191. 10.1111/j.1462-2920.2005.00797.x 16011755

[B143] Tamma P. D. Cosgrove S. E. Maragakis L. L. (2012). Combination therapy for treatment of infections with gram-negative bacteria. Clin. Microbiol. Rev. 25:450. 10.1128/CMR.05041-11 22763634 PMC3416487

[B144] Thormar H. (2010). Antibacterial effects of lipids: historical review (1881 to 1960), in Lipids and Essential Oils as Antimicrobial Agents, ed Thormar H., (Hoboken, NJ: Wiley), 25–45. 10.1002/9780470976623.ch2

[B145] Tomasz M. (1995). Mitomycin C: small, fast and deadly (but very selective). Chem. Biol. 2, 575–579. 10.1016/1074-5521(95)90120-5 9383461

[B146] Turner J. Cho Y. Dinh N.-N. Waring A. J. Lehrer R. I. (1998). Activities of LL-37, a cathelin-associated antimicrobial peptide of human neutrophils. Antimicrob. Agents Chemother. 42:2206. 10.1128/AAC.42.9.2206 9736536 PMC105778

[B147] Verderosa A. Harris J. Dhouib R. Totsika M. Fairfull-Smith K. (2019a). Eradicating uropathogenic *Escherichia coli* biofilms with a ciprofloxacin-dinitroxide conjugate. Med. Chem. Comm. 10, 699–711. 10.1039/C9MD00062C 31191860 PMC6533797

[B148] Verderosa A. D. de la Fuente-Núñez C. Mansour S. C. Cao J. Lu T. K. Hancock R. E. W.. (2017). Ciprofloxacin-nitroxide hybrids with potential for biofilm control. Eur. J. Med. Chem. 138, 590–601. 10.1016/j.ejmech.2017.06.058 28709125

[B149] Verderosa A. D. Dhouib R. Fairfull-Smith K. E. Totsika M. (2019b). Nitroxide functionalized antibiotics are promising eradication agents against *Staphylococcus aureus* biofilms. Antimicrob. Agents Chemother. 10.1128/AAC.01685-19. [Epub ahead of print]. PMC7187575 31636066

[B150] Verderosa A. D. Dhouib R. Fairfull-Smith K. E. Totsika M. (2019c). Profluorescent fluoroquinolone-nitroxides for investigating antibiotic–bacterial interactions. Antibiotics 8:E19 10.3390/antibiotics8010019 30836686 PMC6466543

[B151] Verderosa A. D. Mansour S. C. de la Fuente-Núñez C. Hancock R. E. Fairfull-Smith K. E. (2016). Synthesis and evaluation of ciprofloxacin-nitroxide conjugates as anti-biofilm agents. Molecules 21:841. 10.3390/molecules21070841 27355936 PMC6273952

[B152] Vieira Colombo A. P. Magalhães C. B. Hartenbach F. A. Martins do Souto R. Maciel da Silva-Boghossian C. (2016). Periodontal-disease-associated biofilm: a reservoir for pathogens of medical importance. Microbial. Pathog. 94, 27–34. 10.1016/j.micpath.2015.09.009 26416306

[B153] von Rosenvinge E. C. O'May G. A. Macfarlane S. Macfarlane G. T. Shirtliff M. E. (2013). Microbial biofilms and gastrointestinal diseases. Pathog. Dis. 67, 25–38. 10.1111/2049-632X.12020 23620117 PMC4395855

[B154] Wagner V. E. Iglewski B. H. (2008). *P. aeruginosa* biofilms in CF infection. Clin. Rev. Allergy Immunol. 35, 124–134. 10.1007/s12016-008-8079-9 18509765

[B155] Walters M. C. III. Roe F. Bugnicourt A. Franklin M. J. Stewart P. (2003). Contributions of antibiotic penetration, oxygen limitation, and low metabolic activity to tolerance of *Pseudomonas aeruginosa* biofilms to ciprofloxacin and tobramycin. Antimicrob. Agents Chemother. 47, 317–323. 10.1128/AAC.47.1.317-323.2003 12499208 PMC148957

[B156] Wei G. X. Campagna A. N. Bobek L. A. (2006). Effect of MUC7 peptides on the growth of bacteria and on *Streptococcus mutans* biofilm. J. Antimicrob. Chemother. 57, 1100–1109. 10.1093/jac/dkl120 16595638

[B157] Woehlk H. Trimble M. J. Mansour S. C. Pletzer D. Trouillet V. Welle A. (2019). Controlling biofilm formation with nitroxide functional surfaces. Polym. Chem. 10, 4252–4258. 10.1039/C9PY00690G

[B158] Wood T. K. Knabel S. J. Kwan B. W. (2013). Bacterial persister cell formation and dormancy. Appl. Environ. Microbiol. 79, 7116–7121. 10.1128/AEM.02636-13 24038684 PMC3837759

[B159] Worley B. V. Schilly K. M. Schoenfisch M. H. (2015). Anti-biofilm efficacy of dual-action nitric oxide-releasing alkyl chain modified poly(amidoamine) dendrimers. Mol. Pharm. 12, 1573–1583. 10.1021/acs.molpharmaceut.5b00006 25873449

[B160] Worthington R. J. Richards J. J. Melander C. (2012). Small molecule control of bacterial biofilms. Org. Biomol. Chem. 10, 7457–7474. 10.1039/c2ob25835h 22733439 PMC3431441

[B161] Yang Y. C. Lii C. K. Wei Y. L. Li C. C. Lu C. Y. Liu K. L.. (2013). Docosahexaenoic acid inhibition of inflammation is partially via cross-talk between Nrf2/heme oxygenase 1 and IKK/NF-kappaB pathways. J. Nutr. Biochem. 24, 204–212. 10.1016/j.jnutbio.2012.05.003 22901690

[B162] Yendewa G. A. Griffiss J. M. Jacobs M. R. Fulton S. A. O'Riordan M. A. Gray W. A.. (2019). A two-part phase I study to establish and compare the safety and local tolerability of two nasal formulations of XF-73 for decolonization of *Staphylococcus aureus*: a previously investigated 0.5 mg/g viscosified gel formulation versus a modified formulation. J. Glob. Antimicrob. Resist. 10.1016/j.jgar.2019.09.017. [Epub ahead or print]. 31600598 PMC7136135

[B163] Yepuri N. R. Barraud N. Mohammadi N. S. Kardak B. G. Kjelleberg S. Rice S. A.. (2013). Synthesis of cephalosporin-3′-diazeniumdiolates: biofilm dispersing NO-donor prodrugs activated by beta-lactamase. Chem. Commun. (Camb). 49, 4791–4793. 10.1039/c3cc40869h 23603842

[B164] Yoon B. K. Jackman J. A. Valle-González E. R. Cho N.-J. (2018). Antibacterial free fatty acids and monoglycerides: biological activities, experimental testing, and therapeutic applications. Int. J. Mol. Sci. 19:1114. 10.3390/ijms19041114 29642500 PMC5979495

[B165] Yuan M. Chua S. L. Liu Y. Drautz-Moses D. I. Yam J. K. H. Aung T. T.. (2018). Repurposing the anticancer drug cisplatin with the aim of developing novel *Pseudomonas aeruginosa* infection control agents. Beilstein J. Org. Chem. 14, 3059–3069. 10.3762/bjoc.14.284 30591828 PMC6296412

[B166] Zhao J. Jiang H. Cheng W. Wu J. Zhao J. Wang J.. (2015). The role of quorum sensing system in antimicrobial induced ampC expression in *Pseudomonas aeruginosa* biofilm. J. Basic Microbiol. 55, 671–678. 10.1002/jobm.201300987 25112215

